# Towards realistic privacy-preserving deep learning over encrypted medical data

**DOI:** 10.3389/fcvm.2023.1117360

**Published:** 2023-04-28

**Authors:** José Cabrero-Holgueras, Sergio Pastrana

**Affiliations:** ^1^ Innovation, IT Department, CERN, Geneva, Switzerland; ^2^ Computer Science Department, Universidad Carlos III de Madrid, Madrid, Spain

**Keywords:** privacy-preserving, deep learning, healthcare, homomorphic encryption, SIMD, linear algebra, algorithms

## Abstract

Cardiovascular disease supposes a substantial fraction of healthcare systems. The invisible nature of these pathologies demands solutions that enable remote monitoring and tracking. Deep Learning (DL) has arisen as a solution in many fields, and in healthcare, multiple successful applications exist for image enhancement and health outside hospitals. However, the computational requirements and the need for large-scale datasets limit DL. Thus, we often offload computation onto server infrastructure, and various Machine-Learning-as-a-Service (MLaaS) platforms emerged from this need. These enable the conduction of heavy computations in a cloud infrastructure, usually equipped with high-performance computing servers. Unfortunately, the technical barriers persist in healthcare ecosystems since sending sensitive data (e.g., medical records or personally identifiable information) to third-party servers involves privacy and security concerns with legal and ethical implications. In the scope of Deep Learning for Healthcare to improve cardiovascular health, Homomorphic Encryption (HE) is a promising tool to enable secure, private, and legal health outside hospitals. Homomorphic Encryption allows for privacy-preserving computations over encrypted data, thus preserving the privacy of the processed information. Efficient HE requires structural optimizations to perform the complex computation of the internal layers. One such optimization is Packed Homomorphic Encryption (PHE), which encodes multiple elements on a single ciphertext, allowing for efficient Single Instruction over Multiple Data (SIMD) operations. However, using PHE in DL circuits is not straightforward, and it demands new algorithms and data encoding, which existing literature has not adequately addressed. To fill this gap, in this work, we elaborate on novel algorithms to adapt the linear algebra operations of DL layers to PHE. Concretely, we focus on Convolutional Neural Networks. We provide detailed descriptions and insights into the different algorithms and efficient inter-layer data format conversion mechanisms. We formally analyze the complexity of the algorithms in terms of performance metrics and provide guidelines and recommendations for adapting architectures that deal with private data. Furthermore, we confirm the theoretical analysis with practical experimentation. Among other conclusions, we prove that our new algorithms speed up the processing of convolutional layers compared to the existing proposals.

## 1. Introduction

Cardiovascular diseases produce substantial costs to health systems (1). The invisible nature of these pathologies makes them deadlier and more challenging to track and detect (2). The growing efforts to prevent cardiovascular disease go through continuous and more effective monitoring. However, monitoring and healthcare outside hospitals introduce often-neglected challenges in various domains. First, remote monitoring requires introducing automatic systems that monitor and perform data analytics on patients’ data. Second, due to the sensitive nature of that data, sharing and transmission involve legal, privacy, and security issues.

Deep Learning (DL) has stood as a driver of a new revolution carrying improvements and automation into many fields. In healthcare, multiple examples exist of successful applications of DL (3–5); specifically, these models have succeeded in enhancing medical imaging and health outside hospitals. Deep Learning can help bridge these needs for automatic analysis outside healthcare centers, yet, the sensitive nature of data presents legal and ethical requirements for this data to be shared and adds to existing challenges due to model training and inference and computational capacity (6).

With the recent growth of cloud computing, DL has benefited from significant performance optimizations and flexible environments for its deployment. Concretely, Machine Learning as a Service (MLaaS) allows offloading computations to specialized third-party servers that benefit model owners. This paradigm has two main benefits. First, it relieves the client endpoint from heavy workloads since the processing burden is outsourced to high-performance computing servers (7, 8). Second, it eases the integration of different stakeholders to work in a collaborative environment, where they could contribute with their data to train a common model (9–11). However, scientific applications where sensitive data needs to be exchanged (e.g., healthcare or finances) have not sufficiently profited from the advantages of MLaaS due to ethical and legal restrictions when sharing the data (12, 13).

During the last decade, multiple innovations have enabled secure data exchanges and private computation using Privacy-Preserving Computation Techniques (PPCT), e.g., Homomorphic Encryption (HE) and Secure Multiparty Computation (SMPC) (14). These techniques enhance the security and privacy of the cloud ecosystem by enabling users to safely and privately share data for its computation on external servers, either as a standalone procedure or in a collaborative environment.

**Figure 1:**
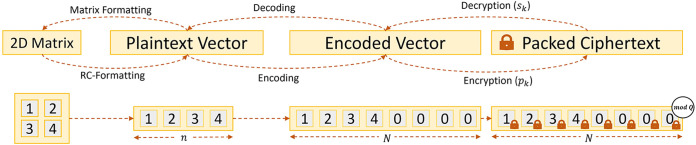
Encryption procedure for Homomorphic Encryption Schemes based on Learning with Errors (LWE).

In particular, HE schemes enable performing operations over the encrypted *ciphertext* without disclosing information about the *cleartext* data. A key milestone was the discovery of bootstrapping by Gentry in 2009, which allows (theoretically) to perform unlimited computation, i.e., HE was proven Fully Homomorphic (15). Since then, multiple improvements have made HE more efficient and practical (16). One such fundamental improvement is *Ciphertext Packing*, which allows the encoding of various entries of plaintext data (encoded as a vector) within a single ciphertext (17). Packing improves efficiency through Single Instruction Multiple Data (SIMD) since the individual operations to the ciphertext affect all the individual entries of the underlying plaintext vector. Figure 1 shows how HE packing works for a 2D Matrix. First, we transform the matrix into a vector using a Row-Column format. Then, we encode (packed) and encrypt it. Then, we can operate the ciphertext can using SIMD, where we modify all the elements of the encrypted vector (matrix)with each instruction.

### 1.1. Motivation

Over the last few years, there has been significant progress on the cryptographic protocols and theories related to ciphertext packing, which promises substantial improvements in the application of HE in complex, distributed applications such as Deep Learning for healthcare remote analysis and monitoring. However, adapting existing operations for SIMD over ciphertext packing is non-trivial.

Unfortunately, the application of ciphertext packing in DL is not straightforward. Indeed, the linear algebra operations used in the internal structures highly affect the performance (18). Understanding the impact of the ordering and election of internal operations on global performance is critical for designing efficient algorithms. However, this complex task requires a proper understanding of cryptographic protocols.

Existing works have attempted to automatically transform linear algebra algorithms so they can be applied using SIMD over packed ciphertexts (18–20) (see Section 6). However, these works provide simplistic views of the required algorithms, which limit their reproducibility and implementation in real-world architectures. Moreover, the algorithms are described and tested for isolated computations, far from the complex workflow and interconnections from the internal layers of Deep Learning. Since the input and output encoding of ciphertext packing differs from regular operations, it is necessary to account for the different formats of the inputs and outputs in each layer of the DL architecture and how these affect the overall performance. For example, when using SIMD operations, it is required to transform the output of a Convolutional Layer to be a valid input for subsequent Dense Layer. These transformations have not been described or accounted for the overall overhead in previous works. Also, depending on specific details of the DL layer, e.g., the use of stride or padding, this transformation requires different adaptions (we provide background on CNNs in Section 2.3). Furthermore, in rotations with packed ciphertexts, the last entries overflow to the first positions. Our work shows how some algorithms profit from this overflow to perform computation.

Overall, existing proposals either leave the adaptation to HE on the user (19) or provide algorithms that are not general for inputs of any size (18). Accordingly, we formulate the following research question:

*How can we adapt linear algebra operations used internally in DL architectures to operate with packed ciphertexts, so they can benefit from the improvements of SIMD operations for Homomorphic Encryption, and thus enable the analysis of privacy-sensitive medical images by untrusted parties?*

### 1.2. Contribution

To tackle the question mentioned above, in this paper we describe a set of algorithms to efficiently transform the operations of Convolutional Neural Networks for their use on packed vectors. A key aspect in the design of the algorithms for cardiovascular diseases is that they work on arbitrary-sized input matrices and vectors, and thus they can adapt to medical images of any size. We provide a holistic view of the DL inference process by not only understanding the individual linear operations required by the algorithms but also the collective relationship of the different layers. For the first time, we show that the transformations required to interconnect the layers pose a significant overhead, which should be considered in the design of the DL architecture.

In summary, the main contributions proposed in this research are the following:

1. We describe algorithms for executing each layer of a Convolutional Neural Network (CNN) using SIMD HE over packed ciphertexts (Section 3). We include algorithms to adapt the input and output encodings to meet the format required on each layer. As a fundamental contribution, we illustrate these algorithms to consider as inputs arbitrary size matrices, allowing their application to different existing architectures. Among these, we provide a streamlined version of the convolution algorithm that significantly improves the efficiency of the previously considered algorithms.
2. We present a formal analysis of the different algorithms and their application to HE (Section 4). We first define a set of metrics to compare the algorithms regarding HE constraints. Then, we analyze the impact of these metrics on the performance of all the algorithms. Finally, using the results of this analysis, we provide a set of recommendations and takeaways for applying the algorithms to DL inference. These conclusions allow us to understand the constraints and challenges of applying SIMD operations inside DL architectures.
3. We empirically test the impact of the proposed algorithms in different use cases to practically verify the takeaways extracted (Section 5). We measure the performance impact of the different stages of the algorithms. We observe how our conclusions match the obtained results. Also, it helps validate our claims that format transformations involve complex processing that we should not take out of the equation for Packed Homomorphic Encryption.

Overall, this paper shows off an existing problem arising from adapting DL architectures’ complex and interrelated operations for their efficient usage with ciphertext Packing and SIMD operations. The proposed algorithms and guidelines will allow programmers to simplify the adaption of existing CNN architectures, thus optimizing the inference process over encrypted data. To assist in the implementation of these algorithms, we provide prototype implementations in our GitHub repository^1^ .

### 1.3. Paper structure

The rest of the paper is organized as follows. First, we provide background information and describe the adversarial model in Section 2. Second, we describe the different algorithms in Section 3. Third, we conduct a formal analysis for the efficiency and performance of the algorithms in Section 4. Fourth, we empirically evaluate a working prototype in well-defined tests in Section 5. Finally, we discuss conclusions and future work in Section 7.

## 2. Background

This section provides base knowledge for the concepts used in the remainder of the document. We first describe the adversarial model and the privacy requirements assumed. Then, we cover an introduction to Homomorphic Encryption, and finally, we describe the Deep Learning Structures considered in the proposed algorithms.

### 2.1. Adversarial model

In this work, we consider an Honest-but-Curious adversary (21–23), a passive adversary that complies with the protocol and does not tamper with the data for malicious purposes. However, it tries to learn as much information from data exchanges. Homomorphic Encryption inherently guarantees input privacy (i.e., the privacy of the data sent to the cloud during the inference process is guaranteed). However, HE does not account for the output privacy of the model. Thus, in our work, we assume that the service provider either is proprietary or has access to the model.^2^

Furthermore, for all of our use cases, we consider the set of parameters established for the Learning with Errors problem following the guidelines described in the Homomorphic Encryption Security Standard (24). Thus, these parameters provide a secure environment for the execution of the algorithms.

### 2.2. Homomorphic encryption

Homomorphic Encryption (HE) is a property of an encryption scheme that permits operating with the ciphertext while translating those changes to the underlying plaintext. This work focuses on widely used HE schemes based on the Ring Learning with Errors (RLWE) problem (25).

Concretely, we focus on Levelled Homomorphic Encryption (LHE) Schemes (16). In these schemes, we represent the RLWE polynomial coefficient modulus Q with a Chinese Remainder Theorem (CRT) coefficient moduli chain $q_{i}$. The representation allows performing a rescaling operation after each multiplication, thus reducing the incurred noise. Also, it reduces the size of the numbers that schemes have to treat. We note that these techniques do not restrict their application to FHE Packed Schemes, but their application to LHE Schemes remains more constraining and thus relevant.

A key point of LHE is the cost of individual operations on the underlying ciphertext representation, as ciphertext are big polynomials (17). Ciphertext Packing arose as a solution by enabling the introduction of more than one plaintext element per ciphertext (17). It permits executing Single-Instructions-Multiple-Data (SIMD) operations (i.e., the execution of an HE operation on a ciphertext propagates to the underlying plaintext vector).

**Figure 2:**
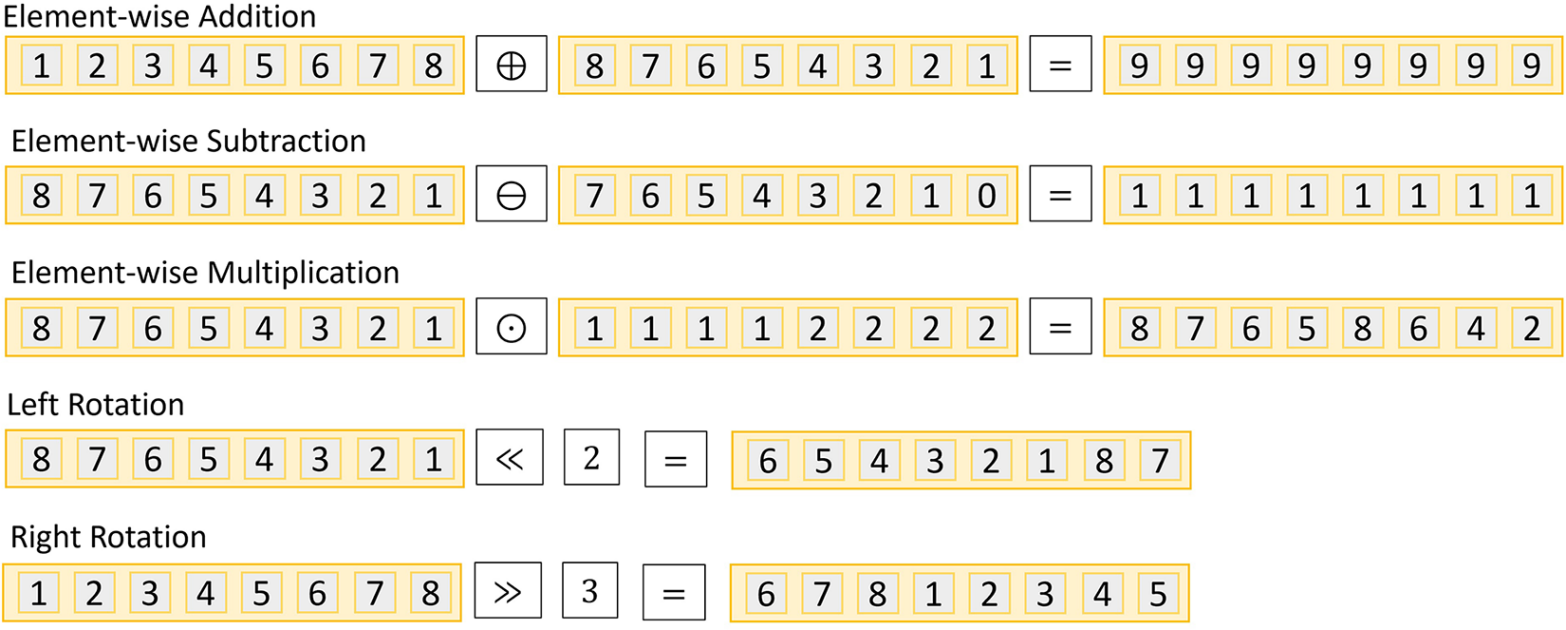
SIMD Operations allowed by CKKS Packed Homomorphic Encryption Scheme and operation scenarios for n=8 and N=16.

An essential aspect of LHE design is parameter selection. The parameter N, or polynomial degree, affects the various matrix operations presented in this paper. This parameter establishes the degree of the polynomial. Also, packed schemes define the number of plaintext elements n that a ciphertext can accommodate, i.e., the maximum length of a vector that can be encoded (see Figure 1). Schemes such as BFV (26) allow packing as many elements as the length of the polynomial (i.e., n≤N). In the scheme CKKS (27), though, due to the complex number packing, it is only possible to pack half of the vector size (i.e., n≤N/2). This paper treats parameter N independently of the scheme (i.e., n=N). However, all the conclusions are valid to CKKS by substituting N by N/2. In Section 4.1, we analyze the implications of different parameter selections on the efficiency of the algorithms. Next, we describe the basic routines of an HE scheme:

∙ A **key generation** routine produces a public key $p_{k}$ and its corresponding private key $s_{k}$, defined by $KG(Q,N)\to p_{k},s_{k}$, where N and Q are the homomorphic encryption parameters.
∙ The **encoding** routine takes a plaintext vector v and encodes it based on the HE parameter N obtaining $v_{enc}$ such that $ENC(v,N)\to v_{enc}$. There is an inverse **decoding** routine that takes the encoded vector $v_{enc}$ and obtains the plaintext vector v such that $DEC(v_{enc},N)\to v$.
∙ The **encryption** routine takes a public key $p_{k}$ and an encoded vector $v_{enc}$ to generate a ciphertext vector $c_{t}\in Z_{Q}[x]/\langle x^{N}+1\rangle$ such that $E(p_{k},v_{enc})\to c_{t}$. The inverse **decryption** routine takes a ciphertext $c_{t}$ and uses the private key $s_{k}$ to obtain the encoded vector such that $D(s_{k},c_{t})\to v_{enc}$.
∙ The different **evaluation** routines compute over the ciphertext $c_{t}$ one of the following operations: Element-wise Sum (⊕) Element-wise Subtraction (⊖), Element-wise Multiplication (⊙), and left/right Rotation (≪ and ≫ respectively). We depict the SIMD schemes operation behavior in Figure 2.

### 2.3. Convolutional deep neural networks inference

Deep Learning is a set of statistical algorithms based on Deep Artificial Neural Networks. These algorithms have shown proficiency when learning from large amounts of data (6). The basic building blocks of DL models are layers (i.e., the different arrangements of neurons in an architecture). Each layer operates with the data differently, depending on its type (e.g., convolution or dot-product). The basic building blocks of layers have been ported to hardware acceleration and multiple libraries (28–30). This work focuses on Convolutional Deep Neural Networks (CNN). These are nowadays the most prominent networks to deal with images (3, 31) (e.g., classification, object detection, or segmentation). Thus, the use of CNN with privacy-preserving techniques is of paramount importance. For further information on CNN, we refer the reader to the book by Goodfellow et al. (32). Next, we overview the basic structures that require adaptation to work with SIMD operations.

*Convolutional layers* compute the correlation between a multidimensional input matrix and a kernel or filter. When dealing with 2D images, we consider it to be the relation between an input image of dimensions $M\in R^{h\times w\times c}$ and a set of kernels or filters $F\in R^{\;f_{x}\times f_{y}\times c^{\prime }}$. The convolution iterates through regions of size $\left(f_{x},f_{y}\right)$ and computes the convolution with each filter F (i.e., multiplication and sum). Usually, in combination with convolutional layers, padding, and stride mechanisms are paired. Considering a picture over which we place a filter, the stride defines the axial displacement of the filters ($s_{x},s_{y}$). Padding a matrix consists of uniformly adding a value p times at the beginning and end of each dimension. In CNN, padding highlights potential features existing on the borders and corners of the image that otherwise would disappear.

*Pooling layers* act as aggregation component. They reduce the size of previous layers and help keep the number of parameters low. They perform a similar operation to the convolution, taking ($p_{x},p_{y}$) regions of the input 2D matrix $M\in R^{h\times w}$ and computing an operation over the region. The most common type of pooling layer is the max pooling layer. It involves computing the maximum pixel of the extracted region. Unfortunately, the max function is non-linear. With current LHE schemes, non-linear functions require polynomial approximations (e.g., Taylor or Chebyshev approximations (33)), incurring extra complexity. Thus, it is also possible to use another classical, yet-effective approach to the Average Pooling Layer, which applies the following operation:

$$\frac{1}{n}*\sum_{i=x}^{x+p_{x}}\sum_{\;j=y}^{y+p_{y}}M_{i,j}$$

*Fully connected or dense layers* link each input element to all output activations. Dense layers usually translate to matrix multiplication between weights matrix ($W\in R^{h\times w}$) and the input vector ($x\in R^{w}$) and adding a bias element ($b\in R^{h}$) (i.e., z=Wx+b). In the weights matrix, each entry represents the relation between the input element y and output element x (i.e., each row relates to an input element, and the specific column represents the relationship with the output element).

*Activation functions* are often non-linear functions that involve introducing non-linear behaviors in the approximated functions. The rationale of these functions is to resemble synaptic signals transmitted by biological neurons (34). Thus, without these, Neural Networks would reduce to complex polynomials. In this work, we do not cover activation function algorithms, as they involve operating on all entries of the ciphertext (i.e., it is straightforward with SIMD). Regarding the existing approximations of non-linearities, we refer the reader to different works (35, 36).

### 2.4. Privacy-preserving deep learning inference

In this paper, we consider a use case where a client-server scenario, where a client needs to outsource the computation of DL inference to a not necessarily trusted server. For that, the client and server rely on public-key FHE. The client performs the key generation, encrypts the data with his public key, and sends the ciphertext to the server. The server receives the public and relinearization keys and the ciphertext. Through privacy-preserving processing, the server can operate on the data and return the result to the client. The client owns a private key which he never released, therefore is the only person able to decrypt the information. Also, we consider that the client performs no computation except light tasks before encrypting or after decryption. For example, the client can perform padding to reduce the load of convolutions since it is a light task. Also, after decrypting the information, the client can retrieve the relevant information entries instead of having the server post-process the result.

## 3. SIMD algorithms for deep learning

Most Deep Learning building blocks rely on standard linear algebra operations (e.g., matrix or vector multiplication). Some of these operations are not available in the encrypted domain (e.g., accessing an arbitrary entry of an array). Furthermore, existing optimizations for running classical linear algebra on computers, such as tiling memory accesses in matrix multiplications (37), are not possible when the smallest unit considered is a packed HE ciphertext (i.e., a polynomial in $Z_{Q}[x]/\langle x^{N}+1\rangle$). Thus, it is necessary to develop focused algorithmic optimizations for these ciphertexts.

In this section, we provide general algorithms to adapt linear algebra operations so they can exploit the potential of SIMD operations in Deep Learning while working on HE ciphertexts. Concretely, we propose descriptions of algorithms that work on arbitrary matrices $M\in R^{h\times w}$ (i.e., of height h and width w).

Additionally, since DL architectures consist of connected layers of different natures, the representations between these layers need to be compatible. This compatibility means that the output of SIMD operations resulting from a given layer needs to be formatted according to the input of the following layer. While previous works have proposed SIMD operations for these layers, they have only provided partial examples, not giving a holistic view of the DL pipeline and not considering the interconnections of the layers (18, 19). Indeed, as we show in Section 5, the data transformations have a considerable overhead which the DL design phase should account for.

When dealing with Packed Homomorphic Encryption, the ciphertext encodes vectors of size N. However, CNNs operate over matrices which require transforming matrices into vectors (see Figure 1). We consider a standard representation that we refer to as Row-Column (RC) format, where the 2D matrix is flattened row by row (Equation 1). This format allows the representation of information with the smallest N. Accordingly, in this work, we provide algorithms to transform the RC format to the appropriate Initial Representation for the algorithm and Result Transformation algorithms for returning to the RC format

(1) $$RC(M)=\{M_{0,0},M_{0,1},\ldots ,M_{0,w-1},M_{1,0},\ldots ,M_{h-1,w-1}\}.$$

In a nutshell, the processing of each layer requires the following algorithms (executed before, during, and after the actual data processing):

1. **Initial Representation (IR)** algorithms provide the corresponding layer with an appropriate representation of the data for executing the algorithm (i.e., according to the requirements of the layer).
2. **Algorithm Execution (ALG)** algorithms are the actual execution of the internal operations over the data. We next describe the convolution blocks (i.e., convolutional layer, pooling layer, and activation function) and dense blocks (i.e., dense layer and activation function).
3. **Result Transformation (RT)**. Due to the nature of SIMD operations, the algorithms usually introduce some extra, irrelevant data in the result (e.g., redundant or padded). Also, different SIMD operations produce different output formats. Thus, we elaborate dedicated algorithms to extract the relevant output information and turn it back into a format suitable for the next layer. We note that this process (RT) can sometimes be computed together with the Initial Representation (IR) of the following layer.

### 3.1. Notation

We use $M\in R^{h\times w}$ to represent a matrix M of dimensions h×w. Also, we use $M_{i,j}$ to refer to the entry on row i and column j of the matrix.

In Section 2.2, we described the encoding, encryption, decoding, and decryption routines. For simplicity, in the algorithms, we assume that a vector’s encryption routine also comprises the previous encoding. Similarly, the decryption routine comprises the decoding after decryption. Thus, we denote $c_{t}=E(p_{k},M\in R^{h\times w})$, as the encryption of the encoded matrix M, whose layout in the encrypted vector may be defined depending on the algorithm.

Many of the algorithms rely on binary bitmasks to obtain relevant information. These are composed of binary values. In the description of the algorithms, we assume bitmasks are initially filled with 0, and we express a condition to get the positions (indexes) where entries are set to 1. We use the parameter t ($bitmask[t]\in Z_{2}^{N}$), which defines such indexes. For example, for a bitmask where even indexes are 1, we denote the mask as bitmask[t]←{tmod2=0}.

In HE, the ciphertext representation uses integer ring polynomial representation, unlike Deep Learning floating point representation. This problem has multiple approaches, such as fixed point representations or CKKS encoding (27). The algorithms provided here are represented generically without discussing the specific representation (i.e., the only requirement is the availability of the operations defined in Section 2.2).

**Figure 3:**
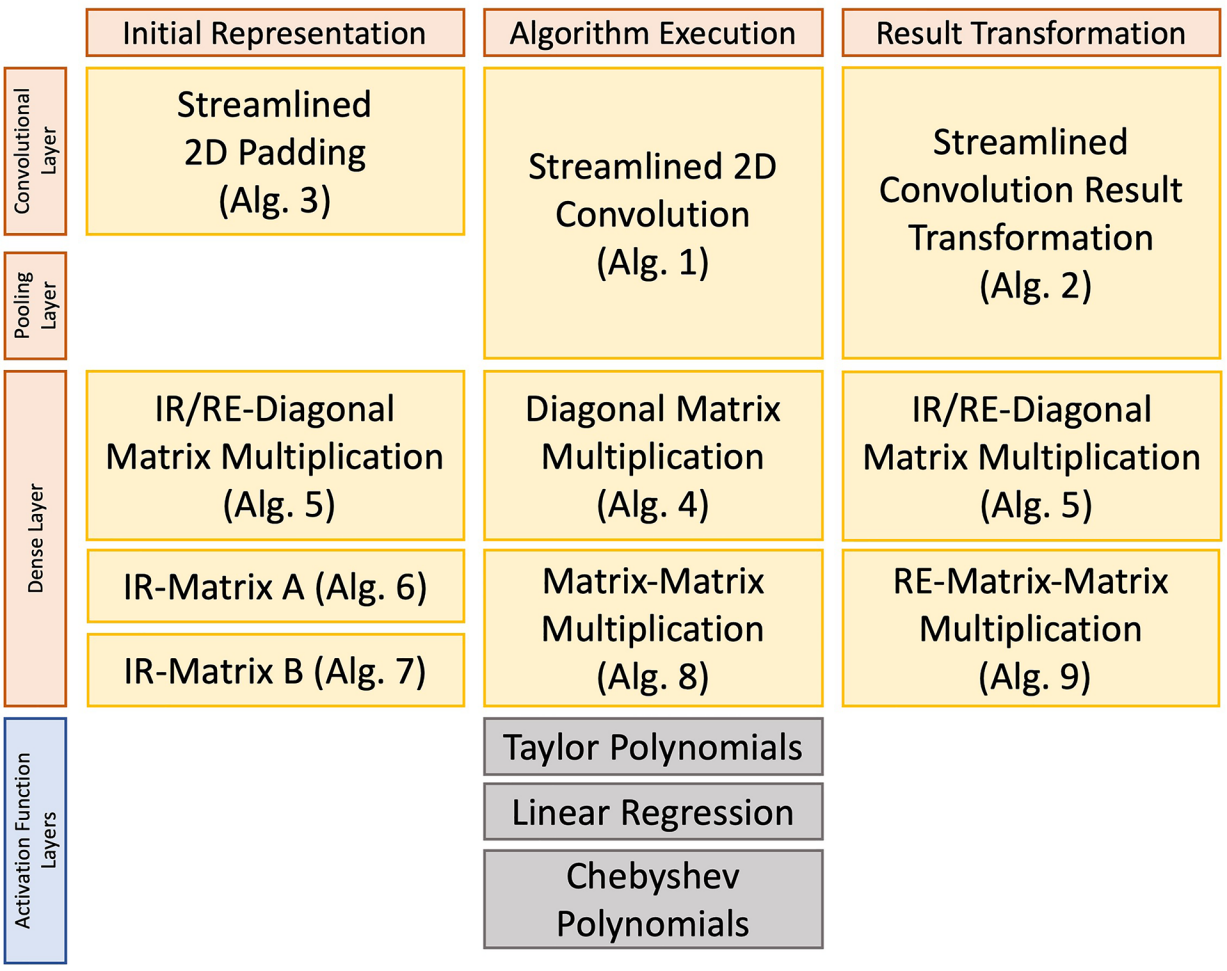
Overview of the different algorithms needed to perform a Convolutional Neural Network with Homomorphic Encryption.

In the following sections, we provide the general algorithms that allow the adaptation of common layers in CNNs, i.e., Convolutional and Dense blocks. Then, we provide a discussion on how to use activation functions (which are non-linear) in the context of Packed Homomorphic Encryption. Figure 3 provides a summary overview of the application of the different algorithms in a CNN pipeline.

### 3.2. SIMD convolutional layer

Computing a classical 2D Convolutional Layer involves a relationship between an input bi-dimensional matrix region and a bi-dimensional filter. As explained in 2.3, the convolution can combine structures such as padding, stride, or pooling layers. Accordingly, the algorithms defined for LHE should also account for the use of these variants.

This section presents a new algorithm for convolution, dubbed the Streamlined Convolution Algorithm. It allows combining convolutional, pooling, and activation layers in subsequent blocks, neglecting the cost of initial representation and executing a single result transformation algorithm (i.e., we can use the output representation of the algorithm arbitrarily). We first describe the convolution algorithm with stride and the result transformation algorithm (Section 3.2.1). Then, we provide its integration with padding (Section 3.2.2). and Average Pooling Layers (Section 3.2.3. For reference, Appendix A details the convolution algorithms that extend and generalizes previous work (18), which we use as a baseline comparison in Section 5.

#### 3.2.1. Streamlined convolution algorithm

The convolution algorithm takes as input a plaintext filter $F\in R^{\;f_{x}\times f_{y}}$ of dimensions $\;f_{x}\times f_{y}$. The filter is applied to a ciphertext vector $c_{t}\in Z_{Q}[x]/\langle x^{N}+1\rangle$ that corresponds to an encrypted input matrix, i.e., $c_{t}=E(p_{k},M\in R^{h\times w})$, with $p_{k}$ being the encryption key and M the plaintext input matrix linearized. The algorithm leverages that the dimensions of filters are shorter than input matrices, and we have plaintext access to those. Thus, it computes the convolution between each filter pixel and the input matrix (i.e., represented by a ciphertext) and adds the partial results for each pixel. The algorithm is described in Algorithm 1.

**Algorithm 1:** Streamlined 2D Convolution

**Input:** $c_{t}^{SCBF}\in Z_{Q}[x]/\langle x^{N}+1\rangle =E(\;p_{k},M\in R^{h\times w})$, $F\in R^{\;f_{x}\times f_{y}}$, $\left(s_{x}^{l},s_{y}^{l}\right)$, p, $\left(S_{x},S_{y}\right)$, $h_{0},w_{0}$ **Output:** $conv\in Z_{Q}[x]/\langle x^{N}+1\rangle$ in SCBF format **function** CONVOLUTION ($c_{t}$,F, $s_{x},s_{y}$, p, $S_{x},S_{y}$, $h_{0},w_{0}$) $h_{out}\leftarrow \lfloor \frac{h-f_{x}+2\cdot p}{s_{x}^{l}}\rfloor +1$ $w_{out}\leftarrow \lfloor \frac{w-f_{y}+2\cdot p}{s_{y}^{l}}\rfloor +1$ $c_{t}^{\;pad}\leftarrow \text{P}\mathrm{ADDING}(c_{t}^{SCBF},p,(S_{x},S_{y}),(h_{0},w_{0})$ **for** $i\leftarrow 0,f_{x}$ **do** **for** $j\leftarrow 0,f_{y}$ **do** $rot\leftarrow c_{t}^{\;pad}\ll (i\cdot w_{0}\cdot S_{x}+j\cdot S_{y})$ $conv=conv⊕rot⊙F_{i,j}$ **end for** **end for** **return** conv **end function**

We denote the linearized format of the matrix as Streamlined Backward Convolution Format (SCBF) since it depends on the information of previous layers. For multiple consecutive convolutions, the algorithm requires some information to determine the layout of the input vector. Concretely, in the execution of layer l, the algorithm receives as input the product of strides from previous layers, i.e., $S_{x}=\prod_{i=0}^{l-1}s_{x}^{i}$ and $S_{y}=\prod_{i=0}^{l-1}s_{y}^{i}$, where $s_{x}^{i},s_{y}^{i}$ are the strides on x and y axis of the ith consecutive convolutional layer. Furthermore, the algorithm also requires the dimensions of the first encoded matrix, i.e., $\left(h_{0},w_{0}\right)$. These define the capacity of the algorithm to perform operations on the information. Whenever we execute a convolution, the result format depends on these values.

For the first layer, we use the Row-Column format, a subset of the SCBF format where $S_{x}=1$ and $h,w=h_{0},w_{0}$.

Finally, if the convolution uses padding, the algorithm relies on the PADDING function, which we describe in the next section.

#### 3.2.2. Streamlined padding algorithm

The insertion of padding is common in convolutional layers to ensure the preservation of details in the corners of matrices when using filters. In general, if the padding is added on the first layer of the CNN, we can apply it on the cleartext matrix (i.e., by the client) and encrypt afterward. However, if the padding is not present on the initial layer, it is the responsibility of the server to execute it. For that, we propose Algorithm 2, where based on the structure of the SCBF format, it performs padding with little computational effort, as opposed to the base algorithm described in Appendix A. The algorithm has two main tasks. First, it rotates the relevant information according to the padding needed for the first row. Then, using a bitmask, it introduces zeroes in the appropriate positions. Note that, for notation clarity, we perform an inversion of the bitmask (i.e., zeroes become ones and vice versa) denoted as 1−bitmask[t].

**Algorithm 2:** Streamlined 2D Padding

**Input:** $c_{t}^{SCBF}\in Z_{Q}[x]/\langle x^{N}+1\rangle =E(\;p_{k},M\in R^{h\times w})$, p, $\left(S_{x},S_{y}\right)$, $h_{0},w_{0}$ **Output:** $c_{t}^{\;pad}\in Z_{Q}[x]/\langle x^{N}+1\rangle =E(\;p_{k},M^{\prime }\in R^{h_{\;pad}\times w_{\;pad}})$, in SCBF format **function** PADDING $c_{t}$, p, $S_{x}$, $S_{y}$, $h_{0}$, $w_{0}$ $h_{\;pad}\leftarrow h+2\cdot p$ $w_{\;pad}\leftarrow w+2\cdot p$ $c_{t}^{\;pad}\leftarrow c_{t}^{SCBF}\gg (w_{0}+1)\cdot S_{x}\cdot p$ $bitmask[t]\leftarrow \{t=i\cdot w_{0}\cdot S_{x}+j\cdot S_{y}|i=\lfloor t/w_{0},j=t\;\mathrm{mod}\;\;w_{0}|i<p\vee i\geq (h_{\;pad}-p)\vee j<p\vee p\geq (w_{\;pad}-p)\}$ $c_{t}^{\;pad}\leftarrow c_{t}^{\;pad}⊙(1-bitmask[t])$ ▷ Inverted bitmask **return** $c_{t}^{\;pad}$ **end function**

#### 3.2.3. Streamlined average pooling layers

Similar to the usage of stride during the convolution operation, Pooling Layers allow for reducing the overall complexity required to process the information. Additionally, they highlight the most relevant features for the classification, extracting higher informative areas and discarding less informative ones. While Max Pooling is a popular choice for Deep Learning since it allows the extraction of pronounced and sharp changes (32) (e.g., edges in pictures for image segmentation), the max function is non-linear. Thus, its usage with current Homomorphic Encryption schemes remains complex and inefficient. In our work, we cover linear average pooling, which results less inefficient,^3^ but extracts smoother changes from pictures (32).

The encrypted version takes an input ciphertext vector $c_{t}\in Z_{Q}[x]/\langle x^{N}+1\rangle =M\in R^{h\times w}$, and a pool $P\in R^{\;p_{x}\times p_{y}}$. The adaptation to SIMD HE can be obtained by using the convolution algorithm presented in Section 3.2.1, but using a dedicated filter for the pooling, defined as:

$$P=\left\{P_{i,j}=\frac{1}{n}|0\leq i<p_{x},0\leq j<p_{y},n=p_{x}\cdot p_{y}\right\}$$

Similar to the convolution algorithm, the pooling layer also requires meta-information from previous layers.

#### 3.2.4. Streamlined convolution result transformation

The previously presented algorithms can be arbitrarily combined between themselves and activation functions, incurring a minimal multiplication depth and the number of multiplications per layer. The only drawback is its combination with other types of layers (e.g., the dense layer). For that, we provide a function allowing the user to return to RC format to become compatible with other layers. The process depicted in Algorithm 3 may not be the most efficient depending on the subsequent layer. If that is the case, the lines detailed as formatting in blue and with the formatting comment may swap for a more appropriate layout. This algorithm receives a ciphertext vector $c_{t}\in Z_{Q}[x]/\langle x^{N}+1\rangle =M\in R^{h_{out}\times w_{out}}$, being the encrypted result of the convolutions of which we stored the output dimensions $h_{out}\times w_{out}$. Additionally, as in the previous examples, it is necessary to keep track of the product of strides in previous layers ($S_{x},S_{y}$) and the initial dimensions ($h_{0},w_{0}$).

**Algorithm 3:** Streamlined Convolution Result Transformation

**Input:** $c_{t}^{SCBF}\in Z_{Q}[x]/\langle x^{N}+1\rangle$ in format, $R^{h_{out}\times w_{out}}$, $h_{0}\times w_{0}$, $\left(S_{x},S_{y}\right)$ **Output:** $c_{t}^{RC}$ in RC format **function** RT-SCR-RC ($c_{t},w_{0},S_{x},S_{y}$) **for** $i\leftarrow 0,h_{out}$ **do** **for** $j\leftarrow 0,w_{out}$ **do** $bitmask[t{]}_{i}\leftarrow \{t=j\cdot S_{y}+(i\cdot w_{0}\cdot S_{x})\}$ $shift_{i}\leftarrow t-(i*h_{out}+j)$ ▷Formatting $c_{t}^{RC}=c_{t}^{RC}⊕(c_{t}^{SCBF}⊙bitmask[t{]}_{i})\ll shift_{i}$ **end for** **end for** **return** $c_{t}^{RC}$ **end function**

### 3.3. SIMD dense layer

The Dense or Fully-Connected Layer of a Neural Network performs a weighted connection of all the inputs to all outputs. It is a linear transformation that a matrix-vector multiplication can represent, where a weight matrix $W\in R^{h\times w}$ is multiplied by the vector $x\in R^{n}$. The weights matrix contains parameters fine-tuned during the training. The input vector comprises all the outputs from the n neurons in the previous layer.

This section proposes algorithms for efficiently applying SIMD operations over encrypted data on Dense Layers. These algorithms generalize two previously proposed algorithms for DL inference: a Diagonal Matrix-Vector multiplication (19) and a Matrix-Matrix multiplication (18). While these matrix multiplication algorithms report simplistic examples, in our work, we describe a generalization together with all the transformation algorithms required for the internal connections.

#### 3.3.1. Diagonal matrix-vector multiplication

This algorithm is based on the multiplication of a ciphertext vector $x\in Z_{Q}[x]/\langle x^{N}+1\rangle$ (i.e., data is encrypted, thus providing input-privacy) by a cleartext matrix $W\in R^{h\times w}$ (i.e., the weights are not encrypted, thus not providing weight-secrecy). The algorithm decomposes the matrix in the extended diagonals (i.e., diagonal vectors of length h). Then the input vector x is rotated, multiplied by the diagonal matrix, and added. In this way, the algorithm ensures that each entry of the ciphertext vector multiplies each matrix value. Halevi and Shoup were the first to describe this approach in HElib (19). In their algorithm, authors do not cover the application to HE where the number of elements in the ciphertext n is fewer than the size of the ciphertext N. To solve this obstacle, we provide an explanation and an Initial Representation algorithm. We also introduce a generalization of the algorithm that enables its application to arbitrary size matrices in Algorithm 4. We discuss the practical implications of its application to HE and its relation to other layers in Section 4.

**Algorithm 4:** Diagonal Matrix Multiplication

**Input:** $c_{t}\in Z_{Q}[x]/\langle x^{N}+1\rangle =E(\;p_{k},v\in R^{n})$, $M\in R^{h\times w})$ **Output:** result **function** DIAGONAL MATMUL ($c_{t},W$) $h_{ext}\leftarrow 2^{\left\lceil log_{2}(h)\right\rceil }$ $w_{ext}\leftarrow 2^{\left\lceil log_{2}(w)\right\rceil }$ $\alpha \leftarrow \max(h_{ext},w_{ext})$ $spacing\leftarrow \frac{N}{\alpha }$ $c_{t}^{ext}\leftarrow \text{S}\mathrm{WITCH}\text{S}\mathrm{PACING}(c_{t})$ **for** i←0,α **do** $d_{i}\leftarrow \{W_{\;j,(i+j\;\mathrm{mod}\;\;w)}|0\leq j<h\}$ $c_{ti}^{ext}\leftarrow c_{t}^{ext}\ll (i\cdot spacing)$ $d_{i}^{ext}\leftarrow \text{E}\mathrm{NC}(d_{i},N)$ $result\leftarrow result⊕(d_{i}^{ext}⊙c_{ti}^{ext})$ **end for** **return** result **end function**

The algorithm takes advantage of the overflow of the input vector during rotations, i.e., when the last values move to the first positions. In HE, the size of the underlying slots is determined by N, which is a power of 2. Introducing a small plaintext vector within a larger ciphertext would prevent us from preserving the overflow behavior. Thus we propose a preprocessing step to keep the overflow happening.

The algorithm relies on two preprocessing steps for correctness. First, to enforce an overflow in longer vectors, the matrix $W\in R^{h\times w}$ is extended to the closest power of 2 in both dimensions, resulting in $\left(h_{ext}=2^{\left\lceil \log_{2}(h)\right\rceil },w_{ext}=2^{\left\lceil \log_{2}(w)\right\rceil }\right)$. Second, based on these new dimensions, a spacing (Δ) is computed to move and split each entry of the plaintext vector uniformly within the ciphertext vector. This way, the shifts can be weighted by the spacing, and the overflow is kept. Concretely, the spacing is computed as follows: $\Delta =N/\max(h_{ext},w_{ext})\in Z$. The denominator takes the maximum since a matrix multiplication either reduces or increases the size of the matrix. Note that since N is a power of 2, and so are $h_{ext}$ and $w_{ext}$, the result is an integer spacing value, also a power of 2. Algorithm 5 shows the algorithm for these preprocessing steps. The algorithm is adapted to switch from a $\Delta_{i}$ spacing between vector elements to $\Delta_{f}$ spacing. In this way, the algorithm can be used both as the Result Transformation of a Dense Layer and the Initial Representation of a subsequent Dense Layer.

**Algorithm 5:** Initial Representation and Result Transformation for Diagonal Matrix Multiplication

**Input:** $c_{t}\in Z_{Q}[x]/\langle x^{N}+1\rangle =E(\;p_{k},v\in R^{n})$, $\Delta_{i}$, $\Delta_{f}$ **Output:** $c_{t}^{ext}\in Z_{Q}[x]/\langle x^{N}+1\rangle$ **function** SWITCHSPACING ($c_{t},\Delta_{i},\Delta_{f}$) $shift=\Delta_{f}-\Delta_{i}$ **for** i←0,n **do** $bitmask[t{]}_{i}\leftarrow \{t=i*\Delta_{i}\}$ $c_{t}^{ext}\leftarrow c_{t}^{ext}⊕((bitmask[t{]}_{i}⊙c_{t})\gg (i*shift))$ **end for** **return** $c_{t}^{ext}$ **end function**

#### 3.3.2. Matrix-matrix multiplication

We propose an algorithm for a matrix-to-matrix multiplication, which takes as a starting point an example provided in CHET for matrices of 3×3 (18). Besides proposing a general description to apply this in arbitrary size matrices, we also provide the Initial Representation and Result Transformation algorithms so these matrix multiplications can be chained together in a DL architecture.

We assume that we want to multiply two matrices $A\in R^{h_{A}\times w_{A}}$ and $B\in R^{h_{B}\times w_{B}}$, which are encrypted and formatted in Row-Column format, $c_{t}^{A}\in Z_{Q}[x]/\langle x^{N}+1\rangle =E(p_{k},A\in R^{h_{A}\times w_{A}})$ and $c_{t}^{B}\in Z_{Q}[x]/\langle x^{N}+1\rangle =E(p_{k},B\in R^{h_{B}\times w_{B}})$. The key concept for the algorithm is the replication of the RC matrix representation. These special and alternative replications permit linear computation of all the necessary combinations. Thus, the main complexity of the algorithm resides in the Initial Representation algorithms. Once this is completed, the overall multiplication complexity is very low.

For matrix $A=\{A_{i,j}|0\leq i<h_{A},0\leq j<w_{A}\}$, its placement over the vector is repeated alternatively according to the formula $c_{t}^{PA}=\{c_{t_{k}}^{PA}=A_{i,j}|1\leq i<h_{A},1\leq j<w_{A},0\leq k=\lceil \left(i+j\cdot w_{A}\right)/w_{B}\rceil <h_{A}\cdot w_{A}\cdot w_{B}\}$. Thus, *each element $A_{i,j}$ of matrix A is consecutively repeated $w_{B}$ times in the vector representation $v_{A}$* (e.g., for A=[1,2,3], $c_{t}^{PA}=[1,1,\ldots ,2,2,\ldots ,3,3,\ldots ]$). Algorithm 6, shows the process to prepare the matrix $c_{t}^{PA}$ from a Row Column representation of A denoted as $c_{t}^{A}$.

**Algorithm 6:** Initial Representation for Matrix A in Matrix Multiplication

**Input:** $c_{t}^{A}\in Z_{Q}[x]/\langle x^{N}+1\rangle =E(\;p_{k},A\in R^{h_{A}\times w_{A}})$, $w_{B}$ **Output:** $c_{t}^{PA}$ **function** PREPAREMATRIXA ($c_{t}^{A},w_{B}$) **for** $i\leftarrow 0,h_{A}\cdot w_{A}$ **do** $bitmask[t{]}_{i}\leftarrow \{t=i\}$ $c_{ti}^{A}=bitmask[t{]}_{i}⊙c_{t}^{A}$ $partial\leftarrow partial⊕(c_{ti}^{A}\gg (i\cdot (w_{B}-1))$ **end for** **for** $i\leftarrow 0,w_{B}$ **do** $c_{t}^{PA}\leftarrow c_{t}^{PA}⊕(\;partial\gg i)$ **end for** **return** $c_{t}^{PA}$ **end function**

For matrix $B=\{B_{i,j}|1<i<h_{B},1<j<w_{B}\}$, its transformation involves repeating multiple times the vector according to the formula $c_{t}^{PB}\in Z_{Q}[x]/\langle x^{N}+1\rangle =\{c_{t_{k}}^{PB}=$ $B_{\left(k\;\mathrm{mod}\;\;w_{B}),(\lceil k/w_{B}\rceil \;\mathrm{mod}\;\;h_{A}\right)}|0\leq k<h_{B}\cdot w_{B}\cdot h_{A}\}$. Thus, *the Row-Column representation of the matrix B is repeated $h_{A}$ times* (e.g., for B=[1,2,3], $c_{t}^{PB}=[1,2,3,\ldots ,1,2,3]$. Algorithm 7 shows the algorithm to prepare the matrix $c_{t}^{PB}$.

**Algorithm 7:** Initial Representation for Matrix B in Matrix Multiplication

**Input:** $c_{t}^{B}\in Z_{Q}[x]/\langle x^{N}+1\rangle =E(\;p_{k},B\in R^{h_{B}\times w_{B}})$, $h_{A}$ **Output:** $c_{t}^{PB}$ **function** PREPAREMATRIXB ($c_{t}^{B},h_{A}$) **for** $i\leftarrow 0,h_{A}$ **do** $result\leftarrow result⊕(c_{t}^{B}\gg (i\cdot h_{B}\cdot w_{B}))$ **end for** **return** result **end function**

Once both matrices are transformed into the specified layout, the algorithm performs element-wise multiplications of the vectors, obtaining a result vector $c_{t}^{C}=c_{t}^{PA}⊙c_{t}^{PB}$. Finally, it applies $h_{A}$ rotations and sums to the resulting vector, obtaining the final result: $c_{t}^{C^{\prime }}=\sum_{i=0}^{h_{A}}c_{t}^{C}\ll N-(i\cdot w_{B})$ (see Algorithm 8).

**Algorithm 8:** Matrix-Matrix Multiplication

**Input:** $c_{t}^{PA}\in Z_{Q}[x]/\langle x^{N}+1\rangle$, $c_{t}^{PB}\in Z_{Q}[x]/\langle x^{N}+1\rangle$, $R^{h_{A}\times w_{A}}$, $R^{h_{B}\times w_{B}}$ **Output:** $c_{t}^{C^{\prime }}\in Z_{Q}[x]/\langle x^{N}+1\rangle$ **function** MATRIXMATRIXMUL ($c_{t}^{PA},c_{t}^{PB}$) $c_{t}^{C}\leftarrow c_{t}^{PA}⊙c_{t}^{PB}$ **for** $i\leftarrow 0,w_{A}=h_{B}$ **do** $c_{t}^{C^{\prime }}\leftarrow c_{t}^{C^{\prime }}⊕(c_{t}^{C}\gg (N-(i\cdot w_{B}))$ **end for** **return** $c_{t}^{C^{\prime }}$ **end function**

However, due to the nature of the algorithm, this result contains extra spacing that needs to be discarded. Concretely, $w_{B}$ relevant items in the vector (i.e., items from the actual result of the multiplication) are followed by $w_{B}$ non-relevant ones (i.e., irrelevant artifacts). These are discarded in a Result Extraction algorithm to finally get the Row-Column representation of the multiplication (see Algorithm 9, where $c_{t}^{C^{\prime }}$ is the result with spacing that needs to be transformed, and $c_{t}^{A\times B}$ is the output of the transformation).

**Algorithm 9:** Result Extraction Matrix-Matrix Multiplication

**Input:** $c_{t}^{C^{\prime }}\in Z_{Q}[x]/\langle x^{N}+1\rangle$, $R^{h_{A}\times w_{B}}$ **Output:** $c_{t}^{A\times B}\in Z_{Q}[x]/\langle x^{N}+1\rangle$ in RC format: **function** RE-MATRIXMATRIXMUL ($c_{t}^{C^{\prime }}$) $bitmask[t]\leftarrow \{0\leq t<w_{B}\}$ **for** $i\leftarrow 0,h_{A}$ **do** $bitmask[t{]}_{i}\leftarrow bitmask[t]\gg w_{B}$ $c_{ti}^{C^{\prime }}\leftarrow (c_{t}^{C^{\prime }}\ll (i\cdot w_{B}))⊙bitmask[t{]}_{i}$ $c_{t}^{A\times B}\leftarrow c_{t}^{A\times B}⊕c_{ti}^{C^{\prime }}$ **end for** **return** $c_{t}^{A\times B}$ **end function**

### 3.4. Activation functions

Neural Networks have excelled at classification and regression tasks because they can map non-linear distributions. Activation functions are a crucial component of such success, and their integration within FHE schemes is a hot research topic in the literature. Linear approximations of various kinds are among the most successful yet straightforward proposals to introduce activation functions in HE-based DL. Authors have proposed solutions ranging from alternative polynomials (35, 38), Taylor and Chebyshev Polynomials (36) or simple linear regressions (39, 40). The common goal of these works is to obtain a low-degree polynomial to approximate the non-linear behavior as accurately as possible. This paper assumes that the activation functions have somehow been approximated to polynomials using any of the existing proposals. Thus, computing an activation function to Packed Homomorphic Encryption does not require any particular format or construction as it applies the transformation to all the slots of the ciphertext. We can often insert the activation layers with other layers or building blocks of the layers (i.e., algorithm execution and result transformation) without changing the final result. In Section 4.3, we provide insights on where introducing activation functions for the convolutional and dense blocks would be more or less desirable.

## 4. Efficiency analysis of algorithms

The previous section presented algorithms for SIMD execution of CNN inference. In this section, we formally analyze their efficiency and performance impact. Indeed, efficiency is one of the biggest challenges for applying Homomorphic Encryption for Deep Learning. We first define the metrics used to measure efficiency. Second, we provide some insights regarding applying rotations and large ciphertext vectors. Finally, we analyze all the algorithms in terms of the proposed metrics. That enables us to provide a series of guidelines for their application.

### 4.1. Efficiency metrics

Generally, HE operations are performance-wise heavy to execute over ciphertexts. Our analysis focuses on the transformations applied to ciphertexts. Plaintext operations have a negligible impact on the computation; thus, we do not account for them in the analysis. For evaluating these algorithms, we rely on four metrics that define the efficiency of a circuit C:

*Multiplication depth ($D_{C}$)* defines the maximum number of consecutive products that an HE ciphertext needs to apply in a given circuit C. The multiplication depth directly impacts the parametrization of HE schemes, specifically in N and Q. In terms of cleartext operations, N defines the polynomial degree. Thus, a bigger N would involve operating over larger degree polynomials (i.e., more coefficients to compute per ciphertext operation). Also, working with bigger Q involves computing more remainders. In Levelled Homomorphic Encryption Schemes, each multiplication usually requires a rescaling operation to reduce the underlying noise. Therefore, we consider the need for one rescaling per $D_{C}$ (i.e., per multiplication). In this case, we need $D_{C}$ different moduli ($q_{i}$) in a polynomial coefficient modulus Q. In direct relation with Q, N often defines a maximum capacity for a Q (i.e., increasing the $D_{C}$ may not only involve increasing Q but also N). The optimization of these parameters is of paramount importance to obtain better runtimes. For all these reasons, keeping a minimal depth of the circuit is very important for achieving efficiency in the desired computation, which justifies why $D_{C}$ is one of the metrics analyzed for the efficiency of the algorithms.

*Operation cost* differs across the different available computations in HE. Multiplication is the most costly since it not only requires multiplication but is paired with a relinearization phase (i.e., preventing the polynomial degree from growing) and a rescaling phase (i.e., reducing the noise scale). The next more costly operation is rotation, which involves generating different Galois Keys. In CKKS, if the encoding scale s is chosen the same as the smallest modulus prime $q_{i}$, we can neglect the noise and depth cost. Element-wise additions are the lowest cost operation and are considered linear in computation and noise growth. For the rest of the analysis, we denote the addition and subtraction complexity $\left(O_{sum}\right)$ as the number of sums (and subtractions) required by a circuit. Likewise, we consider the multiplication complexity $\left(O_{mul}\right)$ and rotation complexity $\left(O_{rot}\right)$ as the number of multiplications and rotations in the circuit.

*Memory complexity ($O_{mem}$)* accounts for the number of ciphertexts needed in memory to execute one of the algorithms. Given the large memory size of ciphertexts, minimizing the number of ciphertexts simultaneously residing in the main memory is essential.

*Memory constraints ($O_{con}$*) determines the constraints that an algorithm imposes on the size of plaintext vectors n it operates with, so these can fit in ciphertext with N slots (i.e., n<N). If the plaintext vectors do not fit in the ciphertext, the circuit would require an extended vector representation (i.e., the plaintext vectors are packed within multiple ciphertexts). In general, for most algorithms, we consider that for an input matrix $M\in R^{h\times w}$, we can compute the algorithm if h⋅w≤N (i.e., if the full matrix size fits in a ciphertext vector slot). However, some specific algorithm representations of information may define harder or softer limits for the execution. As we detail in the following section, the memory constraints $O_{con}$ directly impact the operation cost. In the following sections, we use r to define the number of ciphertext vectors of size N needed to host a plaintext vector of size n.

### 4.2. Rotations on large ciphertexts

The effect of the “Memory Constraints” $O_{con}$ is important for the Rotation operation. In HE programming, most algebra circuits present memory constraints, given the difficulty of packing all the information within the same ciphertext. In parallelism with classical (non-HE) programming, we could consider when a program does not fit into the available memory and uses swap space. At that point, computation becomes a constraint and is more expensive. In HE circuits with packing, when we need multiple ciphertexts to represent the plaintext data, rotations have a worse impact on the efficiency of algorithms. Indeed, many algorithms rely on rotations to benefit from SIMD operations (e.g., to transform output layouts). Previous works assume rotations as “cost-free” operations and thus use them arbitrarily (18). We observe, however, that when an algorithm is generalized to work on arbitrary-size plaintext inputs (often large scale), the assumption does not hold anymore. Suppose the plaintext vector entries n extend over the available slots N. In that case, multiple ciphertexts are required, and the plaintext vectors and rotation cost are no longer neglectable since at least r=⌈n/N⌉ ciphertext vectors are required.

**Figure 4:**
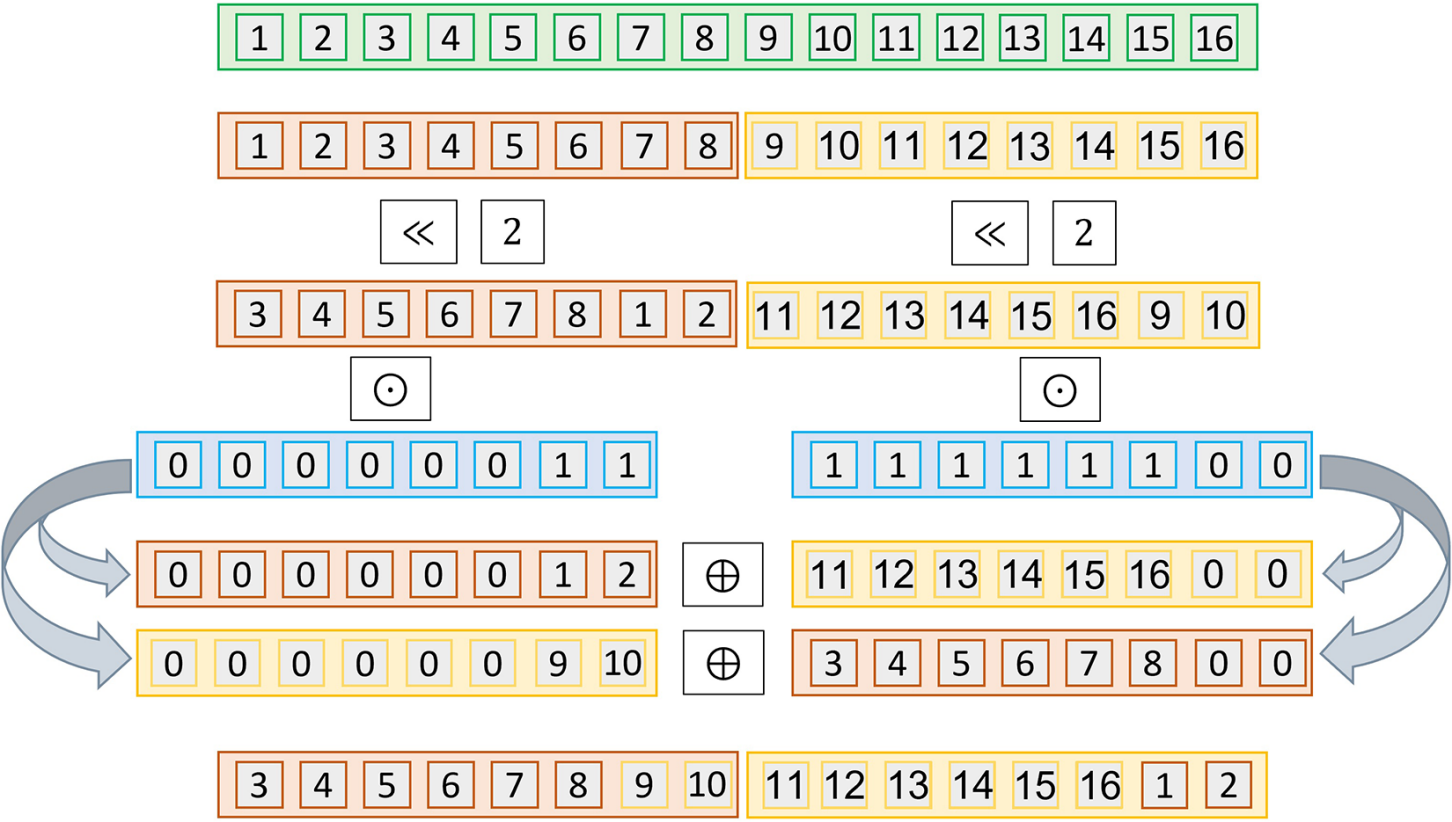
Operations performed for privately rotating twice (ct≪2) a vector of dimension n=16 in with homomorphic encryption ciphertexts of size N=8 and r=2. The vector is encoded in two ciphertexts. The ciphertexts need to be rotated, masked and then reorganized to obtain the same result as in the plaintext rotation.

To demonstrate this performance decrease, we depict in Figure 4 the rotation procedure for n>N, i.e., when more than one ciphertext is needed. In the example, we represent one input vector with two ciphertexts. Considering a 2-left rotation (≪2), we observe that individual rotations of the vectors are partial. Thus, this involves further modifications, such as an additional multiplication of the vectors by a mask, which increases $D_{C}$. Table 1 shows the overall complexity of this process. We provide the details in Algorithm 10.

**Algorithm 10:** Rotation r times of V cleartext vector encoded in multiple ciphertexts $v_{0},v_{1},\ldots ,v_{n}$.

**function** ROTATE ({$\left\{c_{t0},c_{t1},...,c_{tr}\in Z_{Q}[x]/\langle x^{N}+1\rangle \}=E(\;p_{k},M\right)$, rot) q,rot←⌈∗⌉rot/r,rotmodr bitmask[t]←{N−r≤t<N} **for** i←0,r **do** $c_{ti}^{\prime }\leftarrow c_{ti}\gg rot$ $c_{ti}^{0}\leftarrow c_{ti}^{\prime }⊙bitmask[t]$ $c_{ti}^{1}\leftarrow c_{ti}^{\prime }⊖c_{ti}^{0}$ **end for** **for** i←0,r **do** $c_{ti}^{rot}\leftarrow c_{t(i-q\;\mathrm{mod}\;\;r)}^{0}⊕c_{t(i-1-q\;\mathrm{mod}\;\;r)}^{1}$ **end for** **return** $\left\{c_{t0}^{rot},c_{t1}^{rot},...,c_{tr}^{rot}\right\}$ **end function**

**Table 1:** Efficiency analysis of Rotation of a big cleartext vector when it is packed over multiple ciphertexts (Algorithm 10).

Metric	$D_{C}$	$O_{sum}$	$O_{mul}$	$O_{rot}$	$O_{mem}$	$O_{con}$
Value	1	2⋅r	r	r	2⋅r	–

### 4.3. Analysis and takeaways for application to deep learning

In this section, we provide a detailed analysis of the algorithms presented in Section 3 concerning the efficiency metrics proposed. Table 2 presents the performance formal complexity extracted from the different algorithms. Next, we give key insights extracted from the analysis and discuss future directions and best practices to apply the algorithms for Deep Learning Inference.

**Table 2:** Detailed analysis of the different metrics proposed in Section 4.1 ($O_{con}$, $D_{C}$, $O_{sum}$, $O_{mul}$, $O_{rot}$ and $O_{mem}$). Additionally, it shows the performance variation if Rotations are as in Algorithm 10.

Formal analysis of algorithms						
Algorithm	Metrics					
Name	$O_{con}$	$D_{C}$	$O_{sum}$	$O_{mul}$	$O_{rot}$	$O_{mem}$
ALG (1) str. convolution	h⋅w≤N	1	$f_{x}\cdot f_{y}$	$f_{x}\cdot f_{y}$	$f_{x}\cdot f_{y}$	3
	h⋅w>N	2	$3r\cdot f_{x}\cdot f_{y}$	$2r\cdot f_{x}\cdot f_{y}$	$r\cdot f_{x}\cdot f_{y}$	4r
ALG (11) convolution	h⋅w≤N	1	$f_{x}\cdot f_{y}$	$f_{x}\cdot f_{y}$	$f_{x}\cdot f_{y}$	3
	h⋅w>N	2	$3r\cdot f_{x}\cdot f_{y}$	$2r\cdot f_{x}\cdot f_{y}$	$r\cdot f_{x}\cdot f_{y}$	4r
ALG (2) str. padding	$h_{0}\cdot w_{0}\leq N$	1	0	1	1	2
	$h_{0}\cdot w_{0}\leq N$	2	2r	2r	r	3r
ALG (14) private padding	h⋅w≤N	1	h	h	h	3
	h⋅w>N	2	3r⋅h	2r⋅h	r⋅h	4r
RT (3) SCBF to RC	h⋅w≤N	1	$h_{out}\cdot w_{out}$	$h_{out}\cdot w_{out}$	$h_{out}\cdot w_{out}$	3
	h⋅w>N	2	$3r\cdot h_{out}\cdot w_{out}$	$2r\cdot h_{out}\cdot w_{out}$	$r\cdot h_{out}\cdot w_{out}$	4r
RT (12) CRF to RC	h⋅w≤N	1	$h_{out}$	$h_{out}$	$h_{out}$	3
	h⋅w>N	2	$3r\cdot h_{out}$	$2r\cdot h_{out}$	$r\cdot h_{out}$	4r
RT (13) SCRF to RC	h⋅w≤N	1	$h_{out}\cdot w_{out}$	$h_{out}\cdot w_{out}$	$h_{out}\cdot w_{out}$	3
	h⋅w>N	2	$3r\cdot h_{out}\cdot w_{out}$	$2r\cdot h_{out}\cdot w_{out}$	$r\cdot h_{out}\cdot w_{out}$	4r
IR/RT (5) diag. mat. mult.	$n\cdot (\Delta_{f}-\Delta_{i}+1)\leq N$	1	n	n	n	3
	$n\cdot (\Delta_{f}-\Delta_{i}+1)>N$	2	3r⋅n	2r⋅n	n⋅r	4r
ALG (4) diag. mat. mult.	max(α,n)≤N	1	α	α	α	4
	max(α,n)>N	2	3r⋅α	2r⋅α	r⋅α	5r
IR (6) prepare matrix A	$h_{A}\cdot w_{A}\cdot w_{B}\leq N$	1	$h_{A}\cdot w_{A}+w_{B}$	$h_{A}\cdot w_{A}$	$h_{A}\cdot w_{A}+w_{B}$	3
	$h_{A}\cdot w_{A}\cdot w_{B}>N$	3	$3r\cdot h_{A}\cdot w_{A}+w_{B}$	$r(2\cdot h_{A}\cdot w_{A}+w_{B})$	$r\cdot (h_{A}\cdot w_{A}+w_{B})$	4r
IR (7) prepare matrix B	$h_{B}\cdot w_{B}\cdot h_{A}\leq N$	0	$h_{A}$	0	$h_{A}$	2
	$h_{B}\cdot w_{B}\cdot h_{A}>N$	1	$3r\cdot h_{A}$	$r\cdot h_{A}$	$r\cdot h_{A}$	3r
ALG (8) mat-mat. mult.	$\max(h_{A}\cdot w_{A}\cdot w_{B},$ $h_{B}\cdot w_{B}\cdot h_{A})\leq N$	1	$w_{A}=h_{B}$	1	$w_{A}=h_{B}$	4
	$\max(h_{A}\cdot w_{A}\cdot w_{B},$ $h_{B}\cdot w_{B}\cdot h_{A})>N$	2	$3r\cdot w_{A}$	$r(w_{A}+1)$	$r\cdot w_{A}$	5r
RE (9) mat-mat. mult.	n≤N	1	$h_{A}$	$h_{A}$	$h_{A}$	4
	n>N	2	$3r\cdot h_{A}$	$2r\cdot h_{A}$	$r\cdot h_{A}$	3r

#### 4.3.1. The streamlined convolutional blocks reduce the multiplication depth

The improved version of the algorithm we propose in Section 3.2 introduces many efficiency improvements to the base algorithm. The streamlined version of the algorithms allows to insert $l_{conv}$ convolutions, $l_{stride}$ strided convolutions, $l_{\;pad}$ padding layers, $l_{\;pool}$ average pooling layers or $l_{act}$ activation functions (with cost $D_{C}^{act}$). This results in a depth cost of:

$$D_{C}=l_{conv}+l_{stride}+l_{\;pad}+l_{\;pool}+l_{act}\cdot D_{C}^{act}+1$$

The cost is one operation per layer and the Result Transformation algorithm. Also, it does not make any difference in using stridden or non-stridden convolutions. On the other hand, the base version proposed in previous work requires applying a result transformation function after each convolution, and the stride makes it more expensive in the $O_{mul}$. The overall cost of the base version is:

$$D_{C}=2l_{conv}+2l_{stride}+l_{\;pad}+2l_{\;pool}+l_{act}\cdot D_{C}^{act}$$

In summary, the base version heavily affects the depth because it requires result transformation algorithms.

#### 4.3.2. The Streamlined Convolutional Blocks reduce the overall cost of auxiliary convolution routines

If we analyze the cost of operations, we can see how the overall cost of the streamlined convolution algorithm does not change concerning the base algorithm. However, looking at the rest of the streamlined routines (i.e., padding or stride), we can observe how the cost is highly reduced. Although the padding occupies the same multiplication depth slot, it reduces its cost to a single multiplication and rotation. Furthermore, the reduction in the cost of stride permits using it freely, allowing for faster training algorithms over higher dimensionality data. If we used the baseline algorithm, it would be preferable not to use padding and stride to 1 as much as possible to keep efficiency.

#### 4.3.3. Prioritize IR prepare Matrix B over IR prepare Matrix A

Comparing both algorithms, we observe a clear advantage in the algorithm used to prepare Matrix B in the Matrix-Matrix multiplication. Indeed, both $D_{C}$ and $O_{mul}$ are smaller than in Prepare Matrix A. Furthermore, we consider the weights matrix to be provided in cleartext for Deep Learning Inference. In such a case, we recommend prioritizing IR Prepare Matrix A over the cleartext matrix (i.e., executing the heavy algorithm over the plaintext matrix); thus, the IR Prepare Matrix B algorithm on the ciphertext space. It allows for improving the overall performance of the multiplication routine while maintaining input privacy as a constraint.

#### 4.3.4. Avoid using the Matrix-Matrix Algorithm for large input matrices

We observe that the Matrix-Matrix Algorithm (Algorithm 8) imposes significant limits on the size of matrices that can be multiplied ($O_{con}$). For example, for a latent vector size N of 1024, the maximum size of two square matrices A,B that we can privately multiply is around 10×10. Introducing a larger size of N (e.g., 16,384 or 32,768) would improve this factor slightly (e.g., 25×25 and 32×32, respectively). This problem occurs due to the replication factor introduced by the algorithm, i.e., it requires the replication of A’s RC format $w_{B}$ times and B’s RC format $w_{A}$ times. In a real-world setting, Neural Networks often involve larger matrices. Encoding those input matrices often involves using multiple ciphertexts to represent the plaintext vector. The performance would be less in the encoding and execution time (as rotations demand). It also increases the memory requirements and the number of required operations (as described in Table 2).

#### 4.3.5. The Matrix-Matrix multiplication Algorithm improves when B is a one-dimensional vector

This optimization partially overcomes certain of the previously presented weaknesses of this algorithm. Indeed, if we consider a real use case, often Dense Layers are flattened, representing information as a one-dimensional vector. If matrix B is a vector (i.e., $w_{B}=1$), the constraint $O_{con}$ is reduced to $\max(h_{A}\cdot w_{A},h_{B}\cdot w_{A})=\max(h_{A}\cdot w_{A},h_{B}^{2})\leq N$. Furthermore, for reducing Dense layers, where the size of the output vector is smaller than the size of the input vector (i.e., $h_{A}\leq w_{A}=h_{B}$), the constraint would be just on the shape of the underlying vector to the ciphertext. This constraint still imposes hard constraints for the underlying vector size (e.g., around 180 elements for N=32,768 or 128 for N=16,384).

#### 4.3.6. Choosing between Matrix-Matrix or diagonal matrix multiplication mostly depends on $O_{con}$

Table 3 shows both algorithms’ overall cost of an arbitrary l-layer dense architecture. First, it is essential to consider the memory constraints of ciphertexts $O_{con}$. In general, the Matrix-Matrix multiplication remains more efficient for small underlying plaintext vectors n. The improvement is due to having a lower $D_{C}$ and half the number of multiplications $O_{mul}$ than the Diagonal Matrix Multiplication. However, this only holds under the $O_{con}$ assumption, where we can represent the plaintext information under a single ciphertext. If not, the diagonal matrix multiplication becomes more efficient (i.e., the algorithm accepts larger matrix dimensions). At the same time, we must consider that increasing N to permit using more underlying plaintext elements n and working with the Matrix-Matrix multiplication may be counterproductive. This deficiency is due to the cleartext operations performed to execute a ciphertext operation. When we increase N, so does the number of cleartext operations to compute on each polynomial. Therefore, keeping a minimal N becomes likewise critical for efficiency. Before increasing N, using the Diagonal Matrix Multiplication would be better for performance. Finally, if the constraint $O_{con}$ requires multiple vectors in both instances, it would be needed a trade-off between $D_{C}$ and $O_{mul}$ based on the number of layers l. While the overall complexity remains similar for both algorithms, it is important to note two things. First, $O_{mul}$ grows double as the number of layers l grows. Indeed, the Diagonal Matrix multiplication is less efficient for the same number of layers. Second, the increase of $D_{C}$ of l reduces in the Diagonal Matrix Multiplication with a comparison 4l+2≤5l. For neural network architectures with one dense layer l=1, the $D_{C}$ is better with the Matrix-Matrix multiplication algorithm. In the rest of the cases l>1, the overall $D_{C}$ of the Diagonal Matrix is smaller.

**Table 3:** Detailed comparison of the different complete matrix multiplication algorithms described in the paper according to the different metrics proposed in Section 4.1 ($O_{con}$, $D_{C}$, $O_{sum}$, $O_{mul}$, $O_{rot}$ and $O_{mem}$) for a generic l-layer Neural Network. Additionally, it shows the performance variation if Rotations are as in Algorithm 10. Note that, for Algorithms 4 and 5, we can consider α=n. In Algorithms 7, 8, and 9, we consider the optimization of not using Preprocessing A and considering B is a one-dimensional vector.

Matrix multiplication algorithm comparison on l-layer dense neural network							
Algorithm		Metrics					
Name	Alg.	$O_{con}$	$D_{C}$	$O_{sum}$	$O_{mul}$	$O_{rot}$	$O_{mem}$
Diagonal matrix multiplication	5, 4	max(α,n)≤N	2l+1	α(2l+1)	α(2l+1)	α(2l+1)	4
		max(α,n)>N	4l+2	3r⋅α(2l+1)	2r⋅α(2l+1)	α⋅r⋅(2l+1)	5n
Matrix-matrix multiplication	6, 7, 8, 9	$\max(h_{A}\cdot w_{A},h_{B}^{2})\leq N$	2l	$l(2h_{A}+w_{A})$	$l(h_{A}+1)$	$l(2h_{A}+w_{A})$	5
		$\max(h_{A}\cdot w_{A},h_{B}^{2})>N$	5l	$3r\cdot l(2h_{A}+w_{A})$	$2r\cdot l(3h_{A}+w_{A}+1)$	$l\cdot r\cdot (2h_{A}+w_{A})$	6n

## 5. Performance evaluation of guidelines

To study the impact of the algorithms in real-world inference and to corroborate the formal analysis and the critical findings from Section 4, we conduct different experiments. These experiments implement variations from the base use case described in Appendix A (18). In each experiment, we conduct various tests varying the architectures and parameters to examine the performance impact of the different designed routines. All the experiments were run in a computer with processor MD Ryzen 3950X (16 cores at 3.5 GHz), and 32GB of RAM memory.

**Figure 5:**
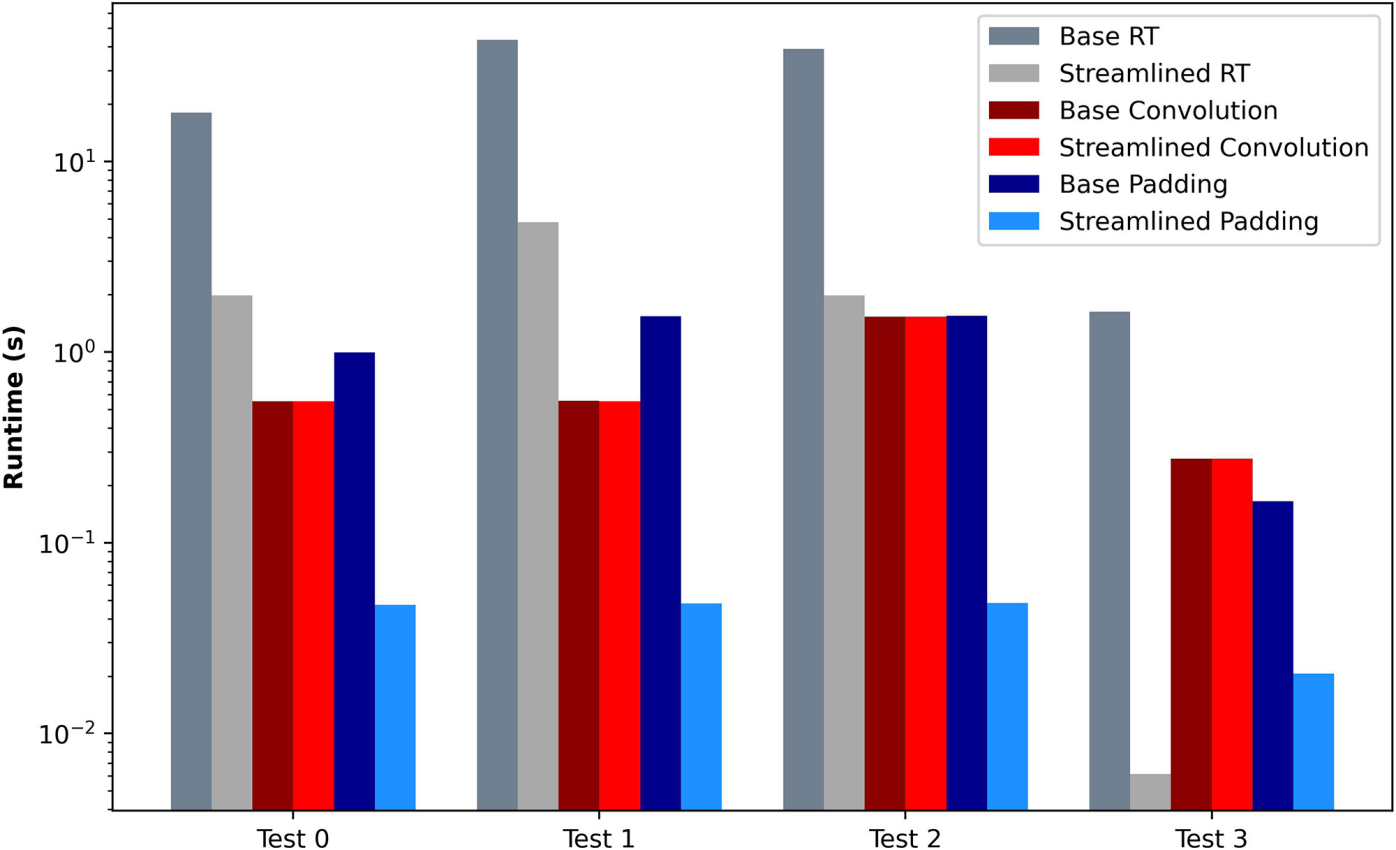
Convolution Performance Evaluation between Streamlined and Baseline approaches to algorithms.

The first experiment compares the baseline and streamlined convolution algorithms. The use case executes a set of c convolutions in a row. All the convolutions have the same properties, with the parameter values described in Table 4. The results are depicted in Figure 5. As expected by the formal analysis, the streamlined convolution algorithm does not impact the convolution operation since the execution times are similar. However, the algorithm produces an output format where the placement of the elements allows for efficient integration with the following layers. It impacts the execution time of the padding and the results transformation algorithms achieving a speedup of 8 times faster on average. Also, we can observe that in the baseline algorithm, the result transformation involves a substantial part of the computational effort, with a high impact on the overall performance.

**Table 4:** Parametrization of the different tests performed for each of the four takeaways considered in Subsection 4.3.

Convolution test case						
Test	Num.	Initial shape	Kernel size	Stride	Padding	Speedup
0	10	20×20	3×3	(1,1)	1	7.51
1	10	30×30	3×3	(1,1)	1	8.37
2	10	40×40	5×5	(1,1)	1	11.75
3	5	30×30	3×3	(2,2)	1	6.86
Matrix-Matrix multiplication test case						
Test	Matrix shape			N		
0	2×2			16		
1	3×3			32		
2	4×4			2048		
3	5×5			2048		
4	20×20			8192		
Diagonal dot multiplication test case						
Test	Matrix shape			N		
0	3×3			16		
1	3×24			64		
2	24×3			64		
3	4×50			2048		
4	50×4			2048		
5	50×50			64		
6	50×50			128		
7	50×75			2048		
8	75×100			2048		
Matrix-Matrix vs diagonal matrix multiplication test case						
Test	Matrix shape			N		
1	5×5			512		
2	10×10			1024		
3	20×20			8192		
4	40×40			65536		
5	50×50			65536		

**Figure 6:**
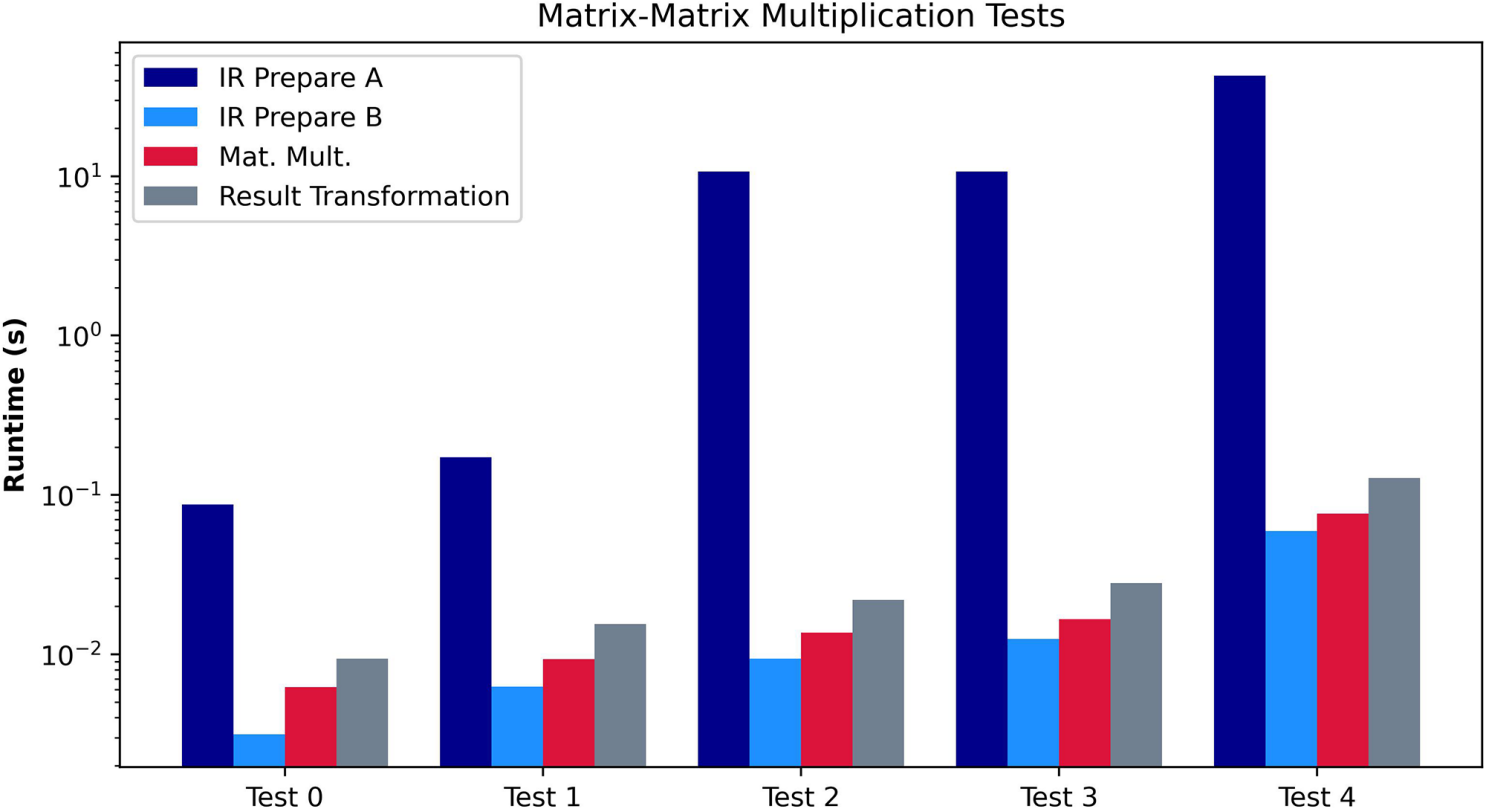
Performance evaluation of Algorithm 8. The graph compares the preprocessing algorithms, Prepare A and Prepare B, in absolute terms, including other subroutines.

In the second experiment, we evaluate the Matrix-Matrix Multiplication algorithm. We compute a sequence of Prepare Matrix Multiplications (for both A and B), the matrix multiplication algorithm, and the result transformation. We show the test cases evaluated in the second section of Table 4 and the results in Figure 6. As demonstrated in Section 4, the IR Algorithm executed for Matrix A is highly inefficient. However, the IR for Matrix B is much more efficient, supposing a relatively minor difference. Therefore, if we consider one of the matrices to be cleartext (e.g., the weights of a Dense Layer are not private), we should always choose it to be matrix A. Another conclusion drawn from Figure 6 is the relevance of the Initial Representation and Result Transformation algorithms. Even in the most optimal use case, the matrix multiplication itself only supposes 40% of the total amount of processing. Therefore, in other works that omit the Initial Representation or Result Transformation, the overall performance is only partially shown, as the transformations involve a significant portion of the overhead.

**Figure 7:**
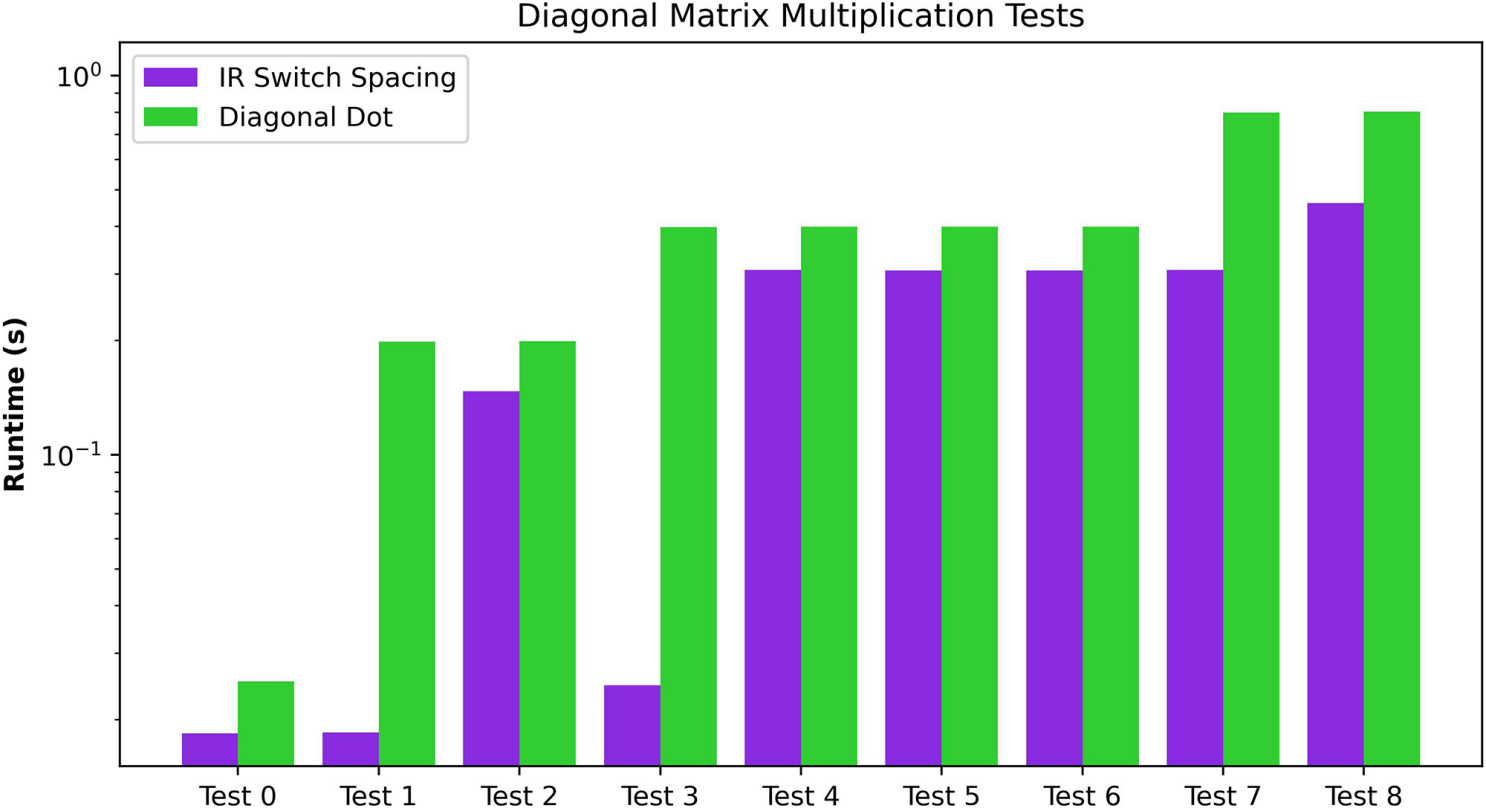
Performance evaluation of the Diagonal Matrix Multiplication of two matrices. The graph shows the overall cost of the two main routines for Initial Representation/Result Transformation and the algorithm in absolute terms and relative to the execution time.

The third experiment analyses the Diagonal Matrix Multiplication. For comparison purposes, we provide a detailed analysis of the diagonal matrix multiplication algorithm in Figure 7. This algorithm generally shows the lesser $O_{con}$ requirements of the Diagonal Matrix multiplications. For bigger matrix sizes, the underlying ciphertext vector needs a smaller size of N than the Matrix-Matrix Multiplication Algorithm. The tests executed on these algorithms can be found in the third subdivision of Table 4. As we can observe, the ordering of dimensions influences the preprocessing algorithm’s time. In general, the maximum dimension of the matrices defines the dimension of the extended diagonal. Therefore, the test with matrices of 50×4 (test 4) obtains similar performance times to the test with matrices of 50×50 (tests 5 and 6). Also, given the small dimensions, the differences between the two tests with matrices of 50×50 (tests 5 and 6) are negligible.

**Figure 8:**
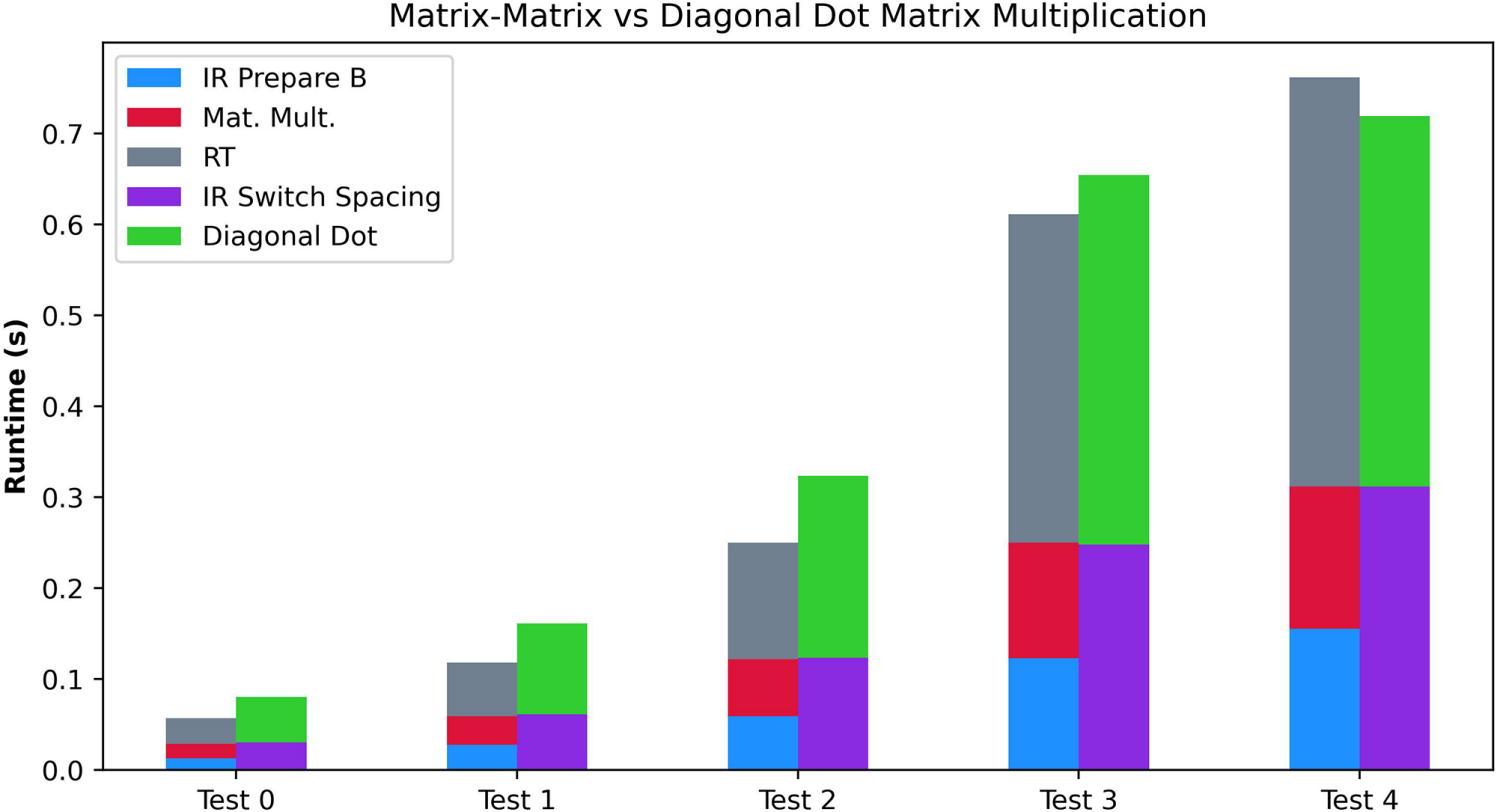
Performance comparison of the Matrix-Matrix Multiplication Algorithm and the Diagonal Matrix Multiplication Algorithms.

The fourth experiment compares the different matrix multiplication algorithms. We perform the fourth experiment with the exact dimensions of the Matrix-Matrix and the Diagonal Dot multiplication algorithms. It enables us to compare the algorithms accurately. We provide the results in Figure 8 and the executed tests at the bottom of Table 4. Overall, we can observe how for smaller sizes of matrices, the lower execution time of the Matrix-Matrix Multiplication Algorithm imposes better runtimes. However, once the dimensions grow, the Diagonal Matrix Multiplication provides better runtimes. However, we note that the tests may give misleading information since, for the same N, the Diagonal Matrix Multiplication enables working with more significant matrices and generally involves less computation.

**Figure 9:**
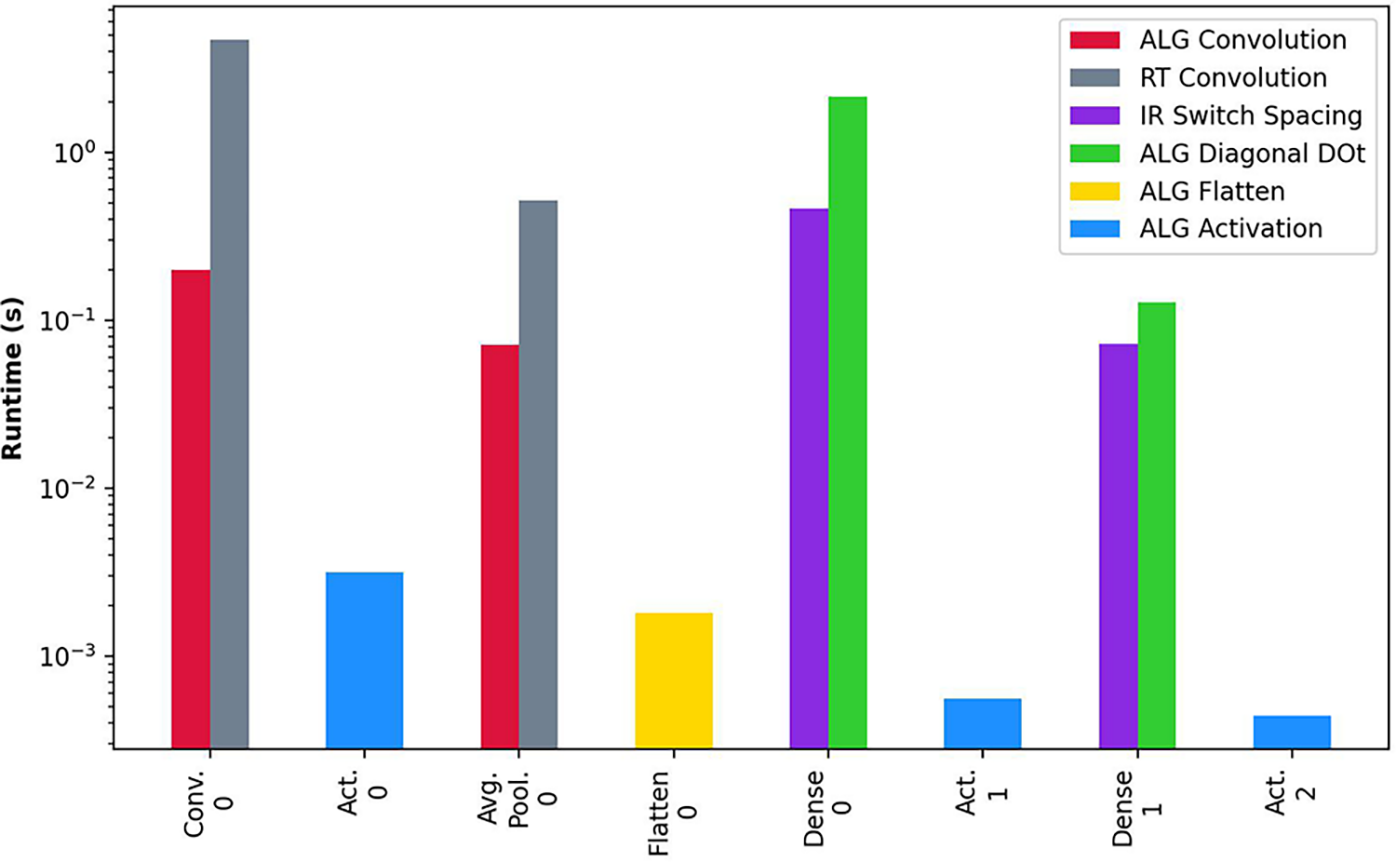
Performance evaluation Cryptonets Neural Network based on the CheXpert dataset. Average runtime of the execution of 250 random samples of the dataset. The ordering of the x-axis corresponds to the layer execution order (i.e., Conv. 0 is the first layer, and Act. 2 is the last layer).

Finally, our fifth experiment combines the different algorithms in a Neural Network use case for cardiology and healthcare. This test analyzes the implications of putting together the algorithm in a real use case. For that, we develop a Convolutional Neural Network model based on the CheXpert dataset (41) with the typical Homomorphic Encryption-based architecture of Cryptonets (35). We perform inference on 250 samples and obtain the average runtime of the layers for the different proposed algorithms. The results of such tests are depicted in Figure 9. First, we observe a noticeable difference between the first layers of the Neural Network and the last layers. As we showed in Section 4, the complexity of the algorithms is often determined by the dimensionality of the treated matrices. The first layers deal with larger dimensions; thus, the computation is more affected by such dimensionality. This fact is especially noticeable with the Convolution and Average Pooling layers, where the Result Transformation is affected by such high dimensionality. Furthermore, when using Homomorphic Encryption, this behavior is emphasized with the existence of LHE, which introduces the concept of levels. Levels are treated with the Chinese Remainder Theorem and operate with more levels before dropping them with each rescaling. On the first layers, the efficiency is worse before rescaling, as HE operates on more remainders than in the latest layers, where most of the moduli have been dropped. Intermediate layers introduce a reduced delay due to being fundamentally a processing-based layer requiring no internal reorganization of the vectors. As a last factor, we analyze the algorithms’ precision compared to classic algorithms and obtain an equivalent absolute precision difference of $3.79\times 10^{-6}$, which we consider negligible for this application.

## 6. Related work

Various works have explored the use of packing within Homomorphic Encryption tasks. With the discovery of FHE by Gentry (15), research on ML tried to discover options for basic statistical models. Wu et al. (39) focus their research on linear regression, mean, and covariance with Somewhat Homomorphic Encryption. Due to the nature of SHE, their work tackles noise management to ensure the operations performed allow correct decryption of the result. The work of Duong et al. (42) goes one step beyond by implementing different embeddings applied when the encoding is performed and used to speed up matrix multiplications. Specifically, they cover two different embeddings, the binary and non-binary, whose placement varies in size and efficiency. These are similar to the replication factors covered in the Matrix-Matrix multiplication algorithm; however, they only cover m×m matrix multiplication, which could often result inefficient.

More recent works have focused on algorithms for Packed SIMD LHE. Halevi and Shoup (19) were the first to propose HE SIMD algorithms in their adaption to HELib (43). In their paper, they provide details on various algorithms, specifically those that allow using HE Packed vectors as usual operations. They cover various algorithms, from individual entry selection to replication and matrix multiplication. In contrast to our work, they provide more general algorithms that consider the minimization of the overload of HE parameters but do not strictly relate them to Deep Learning. Additionally, in our paper, we specifically cover the execution of algorithms for matrix multiplication on arbitrary matrix dimensions, while they only cover square matrices. In our paper, we consider a leveled approach to reduce the overall cost of the circuits in terms of efficiency.

Intel nGraph HE Transformer (44–46) and MP2ML (47) are the first examples of privacy-preserving usability-oriented APIs for DL inference though users still need to incorporate the parametrization manually. Furthermore, the implementations of the different DL routines are opaque.

In CHET (18), the authors proposed a compiler for Deep Learning Inference which considers an analysis of the code to generate efficient HE code. EVA (48) improves CHET parameter selection and reduces it to a multiplication depth parameter. As mentioned before, for the algorithms for Convolution and Matrix Multiplication, we take as a basis the descriptions shown for CHET (18, 48). However, what is shown in CHET is only valid for small matrices. We provide a deeper understanding of the dimensionality of matrices and efficiency guidelines together with the initial representation and result in transformation algorithms.

In GAZELLE (20), the authors propose a framework combining Packed Additively HE with Yao’s Garbled Circuits (49) to speed up DL inference. As part of the packing, they introduce a new method of computing matrix multiplications based on computing halves of the matrix, but whose explanation makes it very difficult to replicate. In our work, we strive for replicability of the algorithms and provide source code for those.

HELayers (50) is a recent framework that aims to provide efficient abstraction layers that fully manage tiling (i.e., the packing of ciphertext vectors, enabling process applications such as DL). In HELayers, the basis for the automatization and interactions between different algorithms is explored.

Finally, Jiang et al. (51) propose a new method to compute matrix multiplication based on multiple iterations where they achieve better efficiency at the cost of a deeper circuit but only cover Matrix Multiplication and not other DL operations.

In comparison to other works, we provide a holistic analysis of the algorithms both individually as an algorithm and collectively within a Deep Neural Network. Furthermore, our analysis through metrics enables us to extract guidelines for use in DL inference. Future works also look in the line of automatic parametrization, combining SIMD algorithms with Homomorphic Encryption for Automated Parametrization (52)

## 7. Conclusions

Classically, healthcare has been limited to treatment in hospitals and health centers. Regulatory and privacy concerns limit the analysis of distributed medical data. It is crucial to allow for secure and efficient sharing and processing of sensitive data, such as health records and medical images, to adapt to the healthcare ecosystem and profit from computational improvements. Deep Learning and Cloud Computing arise as promising game-changing technologies in many fields. However, they still present privacy and security issues related to sensitive data. Homomorphic Encryption introduces a new way to preserve data privacy while performing computation. Not only does it enable secure data sharing and analytics, but it also guarantees privacy, security, and legal implications.

This work describes algorithms to adapt standard linear algebra routines to Packed Homomorphic Encryption (PHE). We focus on operations used in the inference process of Convolutional Neural Networks. In these settings, adapting classical to PHE operations poses additional challenges not covered in previous works. Concretely, our proposed algorithms consider the individual perspective (i.e., the algorithms working in a standalone scenario) and a holistic view (i.e., when different layers work connected in a neural network). A fundamental contribution is the generalization of the algorithms so we can apply them to theoretically arbitrary size inputs. Also, we propose routines such as the *Initial Representation* (IR) and the *Result Transformation* (RT) algorithms to deal with the data format inter-dependencies of the layers.

We elaborate on a set of metrics to represent the impact on the efficiency of PHE operations. The formal analysis of the algorithms shows that their application comes with a cost. Accordingly, we provide analysis for optimal application to DL to adapt existing architectures in the form of key findings. Our experimentation with different tests and use cases shows that the algorithms required to interconnect the layers (i.e., IR and RT) considerably impact the overall performance of the neural network. Thus, we propose optimizations to existing algorithms in the literature that streamline the layers so these additional transformations are optimized. We also provide key findings that can serve during the architectural design of the neural network to optimize its adaption to PHE.

This paper shows practical challenges arising from Packed Homomorphic Encryption with DL, providing a better understanding of the impact of the algorithms. It also provides guidelines for improving algorithms based on efficiency metrics. Also, with this work, we aim to reduce the inherent complexity of the area and understand the base factors upon which data scientists or security experts can design and implement efficient systems. Our experimentation with basic programs and neural networks shows that, in general, the modifications required to the base programs (so they can be used on top of the proposed algorithms) entail negligible computational overhead at no cost in terms of accuracy. This provides a step towards setting up practical, efficient, and private MLaaS services.

Further works should explore the automatic adaptation of algorithms from a classical setting to a vectorized SIMD setting for Homomorphic Encryption, exploiting the relations and the results present in algorithms when performing streamlined operations. Furthermore, establishing tiling frameworks should allow for providing standard representations for input and output, which standardizes the guidelines presented in this paper. Obtaining automatic parameters for Homomorphic Encryption is something that remains relevant as it is one of the more complex tasks in the procedure of Homomorphic Encryption code elaboration.

## Data Availability

The original contributions presented in the study are included in the article/supplementary material, further inquiries can be directed to the corresponding author/s.

## Footnotes

^1^ github.com/anon-dlhe/DeepLearningLinearAlgebra

^2^Output privacy, e.g., preventing model inversion or membership inference attacks, requires mechanisms during training, such as Differential Privacy, which provides privacy protection against adversarial attacks. These are out of this paper’s scope, which focuses on inference and assumes a pre-existing trained model.

^3^We note that so-called hybrid approaches, such as GAZELLE (20), are potential options for evaluating boolean non-linearities. However, they rely on other privacy-preserving computation techniques (e.g., Secure Multi-Party Computation) and, thus, are out of the scope of this paper.

## Appendix A. Baseline convolution algorithms

In this appendix we overview the base algorithms upon which we improve the proposed in Section 3.2. First we describe the base convolution algorithm (Section A.1), its base result transformation (Section A.2 and the introduction of Stride (Section A.3) and padding (Section A.4).

### A.1. Convolution

We propose a general algorithm that permits the application of the convolution operation to arbitrary matrices using SIMD operations. As a starting point, we based the algorithm on examples proposed for matrices of 3×3 using kernel filters of 2×2 (18).

The algorithm takes as input a plaintext filter $F\in R^{\;f_{x}\times f_{y}}$ of dimensions $\;f_{x}\times f_{y}$. The filter is applied to a ciphertext vector $c_{t}\in Z_{Q}[x]/\langle x^{N}+1\rangle$ (in RC format) that corresponds to an encrypted input matrix, i.e., $c_{t}=E(p_{k},M\in R^{h\times w})$, with $p_{k}$ being the encryption key and M the input data in cleartext. The algorithm leverages the fact that the dimensions of filters are shorter than input matrices and that we can operate them in plaintext. Thus, it computes the convolution between each pixel of the filter and the input matrix (i.e., represented by a ciphertext) and adds the partial results for each pixel. The algorithm is described in Algorithm 11.

**Algorithm 11:** 2D Convolution

**Input:** $c_{t}\in Z_{Q}[x]/\langle x^{N}+1\rangle =E(\;p_{k},M\in R^{h\times w})$, $F\in R^{\;f_{x}\times f_{y}}$ **Output:** $conv\in Z_{Q}[x]/\langle x^{N}+1\rangle$ in CRF or SCRF format **function** CONVOLUTION ($c_{t}$,F) **for** {$i\leftarrow 0,f_{x}$ **do** **for** ($j\leftarrow 0,f_{y}$ **do** $rot\leftarrow c_{t}\ll (i*w)+j$ $conv=conv⊕rot⊙F_{i,j}$ **end for** **end for** **return** conv **end function**

Depending on whether we use stride or not, we consider a different result layout. In our work, we name two, the Convolution Resulting Format (CRF) for non-stridden convolution and the Stridden Convolution Resulting Format (SCRF) for stridden convolutions. These layouts include $o_{null}$ meaningless values between the values of the result ($w_{out}$) designated by the input matrix $M\in R^{h\times w}$ and filter size $F\in R^{\;f_{x}\times f_{y}}$. Generally, these formats are not valid for consecutive operations (layers), therefore Result Transformation algorithms are needed, and we describe them next.

### A.2. Result transformation for CRF format

In the CRF format, the amount of $o_{null}$ values is given by $w_{out}=w-f_{y}+1$ and $o_{null}=w-w_{out}=f_{y}-1$. Therefore, a Result Transformation algorithm is developed to transform the CRF format to the Row-Column format (named RT-CRF-RC). The original layout splits the useful rows $w_{out}$ by $o_{null}$ values, therefore, the algorithm creates bitmasks for the rows and shifts them to the appropriate position on the resulting RC format. This processing is described in Algorithm 12.

**Algorithm 12:** Result Transformation CRF to RC

**Input:** $c_{t}^{CRF}\in Z_{Q}[x]/\langle x^{N}+1\rangle$ in CRF format, $R^{h\times w}$, $R^{\;f_{x}\times f_{y}}$ **Output:** $c_{t}^{RC}\in Z_{Q}[x]/\langle x^{N}+1\rangle =E(\;p_{k},M\in R^{h_{out}\times w_{out}})$, in RC format **function** RT-CRF-RC ($c_{t}$, h, w, $f_{x}$ $f_{y}$) $h_{out}\leftarrow h-f_{x}+1$ $w_{out}\leftarrow w-f_{y}+1$ $o_{null}\leftarrow w_{out}-w$ **for** $i\leftarrow 0,h_{out}$ **do** $bitmask[t{]}_{i}\leftarrow \{i\cdot w_{out}\leq t<w_{out}\cdot (i+1)\}$ $row_{i}\leftarrow c_{t}^{CRF}\ll (i\cdot o_{null})$ $c_{t}^{RC}=c_{t}^{RC}⊕(row_{i}⊙bitmask[t{]}_{i})$ **end for** **return** $c_{t}^{RC}$ **end function**

### A.3. Stride

Sometimes, the input matrices to a convolutional layer are high resoultion images (i.e., have long dimensions (h,w)). Processing these images demands high performance cost, since the convolutions extract features from small areas (as defined by the kernel). To avoid processing large portions of the images, the process can be optimized by skipping the result of parts of the convolutions. The amount of data to be skipped is defined by a *stride* tuple $\left(s_{x},s_{y}\right)$. That is, considering that in a normal convolution the output shape is defined by $h_{out}=h-f_{x}+2\cdot p+1$ and $w_{out}=w-f_{y}+2\cdot p+1$, stridden convolutions reduce the output shape by a factor of the stride $\left(s_{x},s_{y}\right)$ such that $h_{out}=\left(h-f_{x}+2\cdot p+1\right)/s_{x}$ and $w_{out}=\left(w-f_{y}+2\cdot p+1\right)/s_{y}$.

Given the convolution algorithm is based on the filter size, the reduction of output elements does not affect the convolution algorithm itself, but it does provide a different layout for the output format. In such a layout, the elements are more scattered than without stride. For reference, we name this layout Strided Convolution Resulting Format (SCRF). As with the CRF format, we cannot directly use the output layout in consecutive layers. Thus, we propose an algorithm that translates from this layout to the RC, dubbed RT-SCRF-RC, described in Algorithm 13. In this algorithm, we use a formula to determine where the $w_{out}$ elements for the stride output are placed and extract them iterative addition through bitmasks.

**Algorithm 13:** Result Transformation SCRF to RC

**Input:** $c_{t}^{SCRF}\in Z_{Q}[x]/\langle x^{N}+1\rangle$ in SCRF format, $R^{h\times w}$, $R^{\;f_{x}\times f_{y}}$, $\left(s_{x},s_{y}\right)$, p **Output:** $c_{t}^{RC}$ in RC format **function** RT-SCRF-RC ($c_{t},h,w,f_{x},f_{y},s_{x},s_{y},p$) $h_{out}\leftarrow \lfloor *\rfloor \frac{h-f_{x}+2\cdot p+1}{s_{x}}$ $w_{out}\leftarrow \lfloor *\rfloor \frac{w-f_{y}+2\cdot p+1}{s_{y}}$ **for** $i\leftarrow 0,h_{out}$ **do** **for** $j\leftarrow 0,w_{out}$ **do** $bitmask[t{]}_{i}\leftarrow \{t=j\cdot s_{y}+(i\cdot w\cdot s_{x})\}$ $shift_{i}\leftarrow t-(i*h_{out}+j)$ $c_{t}^{RC}=c_{t}^{RC}⊕(c_{t}^{SCRF}⊙bitmask[t{]}_{i})\ll shift_{i}$ **end for** **end for** **return** $c_{t}^{RC}$ **end function**

### A.4. SIMD padding

In many modern CNN architectures, it is common to chain multiple convolutional layers. While in the first convolutional layer it is possible to introduce padding in “cleartext” (i.e., the data owner can add it at the end of the ciphertext before encryption), for consecutive ones, it is required to do it privately.

The proposed algorithm takes a bidimensional linearized ciphertext array $c_{t}\in Z_{Q}[x]/\langle x^{N}+1\rangle =E(p_{k},M\in R^{h\times w})$ and pads it uniformly with p zeroes on each dimension. It initially assumes a Row-Column format where the remaining entries of the vector are set to zero. The algorithm extracts each row, and computes the necessary shifting for a row, defined by the formula $shift_{i}=(w+1)+(p\cdot i)|0\leq i<h$. The details are described in Algorithm 14. This algorithm outputs a Row-Column format directly usable by the convolution algorithm described in Section 3.2.1. Furthermore, it does not affect the Result Transformation algorithm.

**Algorithm 14:** 2D Padding

**Input:** $c_{t}\in Z_{Q}[x]/\langle x^{N}+1\rangle =E(\;p_{k},M\in R^{h\times w})$, in RC Format, p **Output:** $c_{t}^{\;pad}\in Z_{Q}[x]/\langle x^{N}+1\rangle =E(\;p_{k},M^{\prime }\in R^{h_{\;pad}\times w_{\;pad}})$, in RC format **function** PADDING ($c_{t}$, p) $h_{\;pad}\leftarrow h+2\cdot p$ $w_{\;pad}\leftarrow w+2\cdot p$ **for** i←0,h **do** $bitmask[t{]}_{i}\leftarrow \{i\cdot w\leq t\leq w\cdot (i+1)\}$ $shift_{i}\leftarrow (w+1)+(\;p\cdot i)$ $c_{t}^{\;pad}=c_{t}^{\;pad}⊕((c_{t}⊙bitmask[t{]}_{i})\gg shift_{i})$ **end for** **return** $c_{t}^{\;pad}$ **end function**
